# Emerging Therapeutic Strategies in Intracerebral Hemorrhage: Enhancing Neurogenesis and Functional Recovery

**DOI:** 10.1002/mco2.70377

**Published:** 2025-10-11

**Authors:** Haojie Zhang, Yinping Pan, Liang Jin, Bochu Wang

**Affiliations:** ^1^ Key Laboratory of Biorheological Science and Technology Ministry of Education, College of Bioengineering, Chongqing University Chongqing China

**Keywords:** hematoma targeting, intracerebral hemorrhage, neurological recovery, regenerative engineering

## Abstract

Intracerebral hemorrhage (ICH) is a serious neurological disease, characterized by a high incidence rate, a high mortality rate, and long‐term neurological dysfunction. Due to the complexity of the pathological mechanism, traditional treatment methods, including surgical intervention and drug therapy, are limited in repairing nerve damage and restoring function. It is necessary to explore innovative treatment strategies. Here, we propose three different pathophysiological stages of ICH, namely, primary injury, secondary injury, and chronic remodeling, and comprehensively discuss the precise targeted treatment of each stage according to the different pathological characteristics. Recent advances in regenerative medicine offer tremendous potential for neurological recovery. This review deeply discusses the emerging biomedical strategies for treating ICH through the integration of cell therapy, intelligent biomaterial platforms, and neuroelectronic interfaces. Furthermore, this review outlines the key clinical translation pathways for emerging therapeutic approaches. By using advanced biomarkers to stratify patients, optimizing combined treatment strategies and overcoming regulatory challenges are of great significance for accelerating the transition of these technologies into clinical practice. This review aims to provide a new perspective for the precise treatment of ICH to improve the neurological prognosis of patients by comprehensively discussing the current research progress and future development directions.

## Graphical abstract

Intracerebral hemorrhage (ICH) causes complex pathophysiological mechanisms such as primary injury and secondary injury, leading to neuronal damage. The emerging treatment strategies can carry out precise treatment at different time stages after ICH, and have offer enhanced targeting, intelligence, and efficiency. These novel approaches have the potential for clinical transformation, aiming to promote neural regeneration and improve the clinical outcomes of patients with ICH.

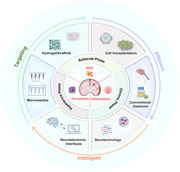

## 1 Introduction

Intracerebral hemorrhage (ICH) is a serious neurological disorder characterized by increased incidence and mortality rates [1, 2]. According to statistics, more than 1.3 million patient die from stroke in China each year, of which ICH accounts for approximately 20–30% [3]. Clinical treatment of ICH has focused on acute phase interventions, such as surgical hematoma evacuation and aggressive blood pressure management, aimed at limiting initial brain damage [4]. Despite these efforts, these approaches have remained insufficient to prevent secondary brain injury or attain good long‐term functional recovery that still leads to disability in patients, placing an enormous burden on society and families [5]. Therefore, there remains a continued clinical necessity for alternative therapeutic options [6].

Treatment of ICH presents considerable difficulties due to its intricate pathophysiological cascade [7]. The primary injury phase involves mechanical compression caused by the hematoma and neurotoxicity of blood components [8]. Subsequently, secondary injury consists of a severe neuroinflammatory response because of activated immunocytes, sustained blood–brain barrier (BBB) destruction, oxidative stress, and excitotoxicity that ultimately cause neuronal deaths [9]. In phase of chronic remodeling, spontaneous neuronal repair and functional circuit reorganization are hindered by glial scar formation and inflammatory response. It is an active process that underscores the crucial importance of treatment windows, and the treatment plan must be precisely timed for selective targeting of pathological phases [10].

To overcome the limitations of traditional therapies, developments of regenerative engineering have driven therapeutic innovation in this field [11]. Emerging strategies now integrate stem cell biology [12, 13], biomedical engineering, and neuroelectronic [14], and can stimulate neural regeneration and restoration by regulating the proliferation of endogenous neural stem cells (NSCs), using cell transplantation to replace dead nerve cells, developing intelligent biomedical platforms, and neural electronic interfaces [15]. Overall, these innovations have revolutionized the treatment of ICH, shifting the focus from alleviating injury to precisely targeting different stages of neural repair.

However, many problems that make it difficult to properly promote these innovative methods from the laboratory to clinical application. It is crucial to propose a reliable clinical translation pathway, and it has the potential to turn the current clinical treatment model into precision medicine by using biomarkers to classify patients’ conditions and implement corresponding personalized treatment measures [16]. In addition, as the complex pathological mechanisms of ICH often require a multitarget treatment approaches, computer simulation platforms can evaluate sequential and concurrent strategies, thereby optimizing combination therapies to enhance therapeutic potential [17]. Finally, the new features of cell therapies and intelligent biomedical platform therapies bring unique and strict regulatory challenges, especially with long‐term safety, teratoma risk, and the degradation kinetics of implanted materials.

**FIGURE 1:**
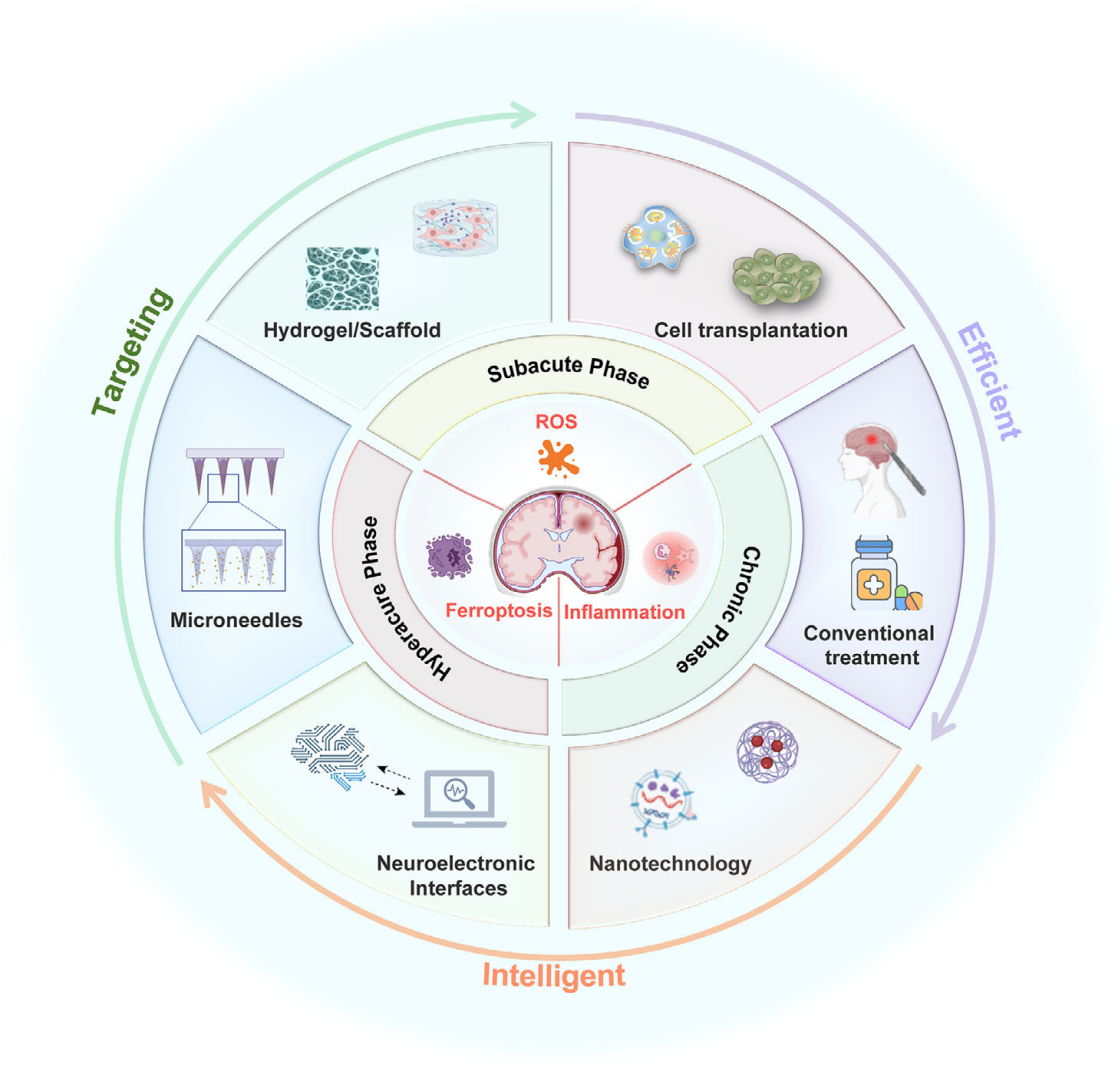
Emerging treatment strategies for ICH promote neurogenesis and functional recovery. ICH initiates a cascade of pathological processes, including oxidative stress, ferroptosis, and inflammatory responses, leading to neuronal injury. The emerging treatment strategies can carry out precise treatment at different time stages after ICH and have offer enhanced targeting, intelligence, and efficiency. These novel approaches have the potential for clinical transformation, aiming to promote neural regeneration and improve the clinical outcomes of patients with ICH.

This review aims to synthesize the latest progress in emerging treatment strategies for ICH, exploring the complicated pathophysiological process and the critical therapeutic windows for intervention in detail. We first describe the different stages of brain injury caused by ICH, and then provide an overview of the specific treatment windows for different stages. The core content of this review focuses on innovative fields of regenerative engineering, including the regulation of endogenous and exogenous cells, smart biomaterial platforms, and neuroelectronic interfaces. Finally, we talk about the crucial clinical translation roadmap, focusing on the utilization of biomarker for patient classification, the optimization of combination treatment strategies, as well as major regulatory challenges associated with these advanced therapies (Figure 1). By integrating insights from various disciplines, this review aims to provide a comprehensive roadmap to accelerate the development and successful implementation of new treatment strategies, ultimately improving the neurological function and quality of life for patients.

## 2 ICH‐Induced Pathophysiological Neural Impairment

**FIGURE 2:**
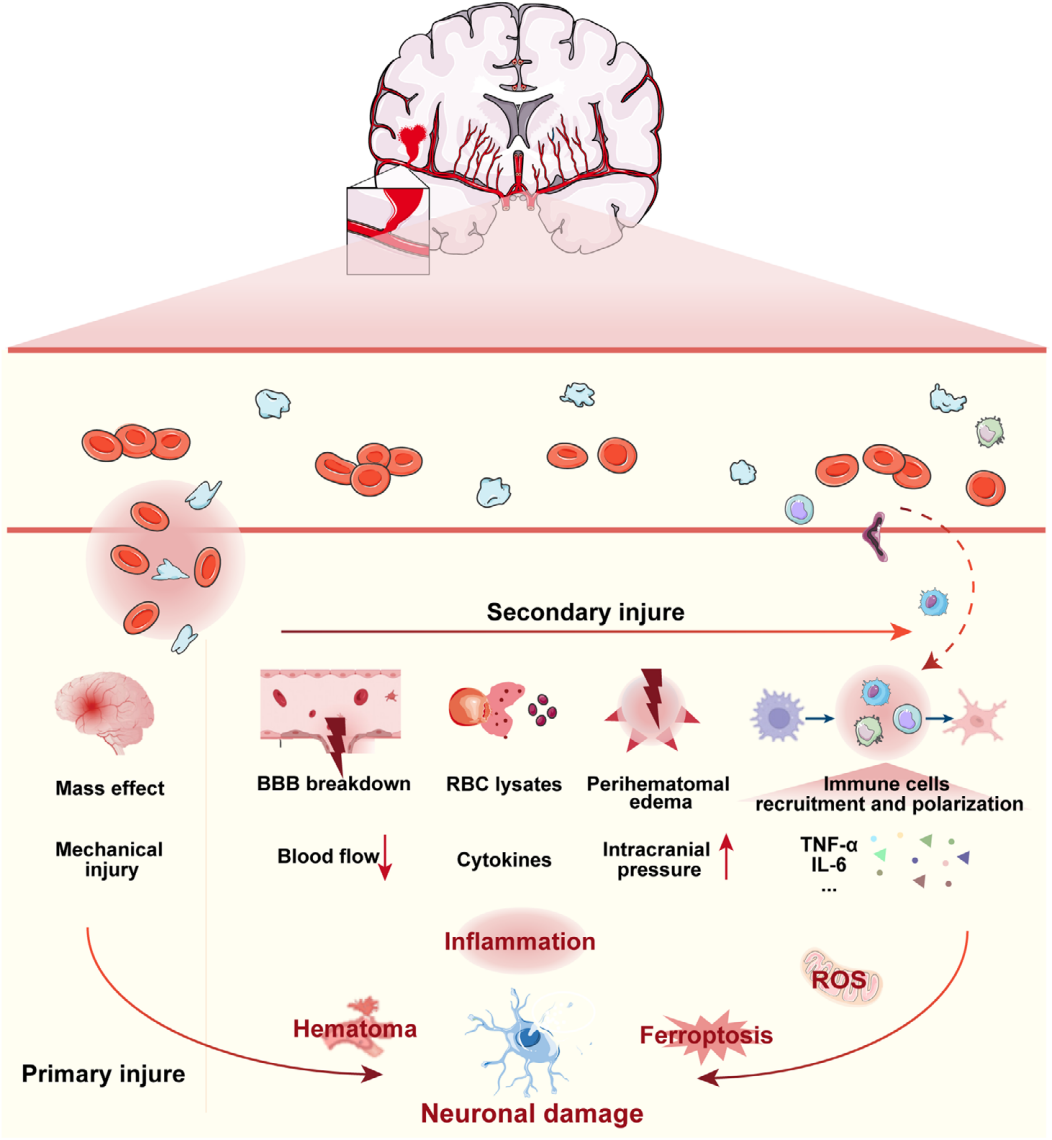
The pathophysiological mechanism of ICH leads to the death of nerve cells. ICH can cause primary injury and secondary injury. Primary injury is the mechanical injury caused by hematoma compression after hemorrhage, followed by secondary injury response characterized by BBB destruction, red blood cell (RBC) lysis, and perihematomal edema, leading to the combined action of multiple pathological processes such as inflammatory response activation, oxidative stress, and ferroptosis cascade reaction. These processes aggravate neuronal damage and significantly affect neurological prognosis.

ICH causes a dynamic and complex pathophysiological cascade, which is usually divided into primary injury, secondary injury, and chronic remodeling [18], representing critical therapeutic windows for intervention and ultimately determining neurological prognosis [19, 20]. Each stage has unique molecular mechanisms, and understanding the progression of disease and mechanisms at each time stage is important to subsequently guide the design of specific treatment measures (Figure 2) [21]. This section will comprehensively outline the key mechanistic characteristics of each stage, laying the foundation for the subsequent discussion of targeted treatment strategies and interventions in a determined time window.

### 2.1 Primary Injury Phase

The primary injury phase of ICH occurs after the rupture of blood vessels and the formation of hematoma. The characterized of acute phase is that brain tissue suffers direct mechanical damage, and the intracranial pressure (ICP) rises rapidly [22]. In addition, the brain parenchyma being exposed to extravasated blood components will trigger a complex cytotoxic reaction [23, 24]. The acute phase is mainly driven by two interrelated but mechanistically different processes, which together lead to subsequent neural damage. The temporal dynamics and spatial distribution of these main injury mechanisms significantly affect the neural recovery process after ICH and provide necessary insights for determining the treatment time window.

#### 2.1.1 Mechanical Compression Effects

Within hours of ICH onset, blood accumulation results in hematoma formation, which leads to increased ICP and applies mechanical pressure to surrounding tissues [25]. Specifically, ruptured blood vessels introduce blood into brain tissue with significant shear force, causing a mass effect and generating a gradient hydrostatic pressure around it, leading to primary injury. Due to the rigidity of the skull and the limited intracranial space, brain tissue is extremely susceptible to various mechanical forces from within the cranial cavity, including shear force from blood, hydrostatic pressure, and mass effect caused by pathological conditions such as cerebral hematoma and edema [26].

The mass effect is a pathological phenomenon caused by brain hematoma and surrounding edema [27]. It is of great significance for the neurologic damage after ICH [28]. The edema around the hematoma can serve as an indicator of the severity of secondary brain injury after ICH and it is also related to the later functional recovery [29]. As ICH progresses, if the hematoma dissolves, the degree of brain edema will increase immediately, making the mass effect more serious. In addition, the toxic substances and mechanical compression produced by the hematoma and blood clots will cause neuronal death, damage the BBB, and aggravate secondary brain damage. Clinical results show that rapid removal of hematoma is beneficial for reducing subsequent secondary brain injury.

#### 2.1.2 Hemolytic Toxicity

Accompanying mechanical damage, the decomposition of RBCs in the hematoma releases hemoglobin, heme, and iron, inducing hemolytic toxicity. RBCs that are not phagocytosed by microglia/macrophages will also undergo lysis, releasing potentially harmful components into the extracellular space [30]. Within the central nervous system, free hemoglobin and its breakdown products are neurotoxic and cause secondary brain damage, leading to the aggravation of brain edema and further damage to the BBB [31]. Free hemoglobin causes damage to neuronal cells and promotes microglia activation via the TLR4 receptor, which can increase the production of proinflammatory cytokines through the NF‐κB signaling pathway [32], and hemoglobin will be further degraded to release heme, which is then metabolized into ferrous/iron under the catalysis of heme oxygenase, inducing reactive oxygen species (ROS) and lipid peroxidation, thereby destroying cell function. In addition, iron can also trigger the release of a large amount of ROS through the Fenton reaction, and iron‐induced oxidation reactions will eventually lead to ferroptosis of neurons [33]. This oxidative stress amplifies neuronal damage and contributes to the transition to secondary injury mechanisms.

When these hematoma components are released into the brain tissue, they activate the complement system and the immune system, including cell lysis and inflammatory responses. Inflammation plays an important role in the destruction of the BBB, and the accumulation of proinflammatory factors released by inflammatory cells will damage nerve cells.

### 2.2 Secondary Injury Phase

Secondary brain injury may result in neurological dysfunction, primarily due to intraparenchymal hemorrhage. Unlike the primary injury, which is immediate and irreversible, the development of secondary brain injury takes hours to weeks and represents a dynamic and potentially modifiable phase of brain injury. ICH can cause cytotoxicity [34], excitotoxicity, oxidative stress, and inflammatory responses [35]. These processes synergistically amplify neuronal damage, exacerbate cerebral edema, and impair the integrity of the neurovascular system.

#### 2.2.1 Neuroinflammatory Storm

As previously mentioned, upon detecting blood components within the parenchyma, inflammatory cells activate microglia and astrocytes [36]. The activation of microglia initiates the immune system's response (macrophages and T lymphocytes), in addition to other cell components like leukocytes, resulting in the release of inflammatory cytokines and the enhancement of local inflammatory processes, including IL‐1β, TNF‐α [37], chemokines, free radicals, and various other inflammatory mediators and toxic substances. Among these, the NF‐κB signaling pathway assumes a crucial role, leading to increased lymphocyte infiltration and continued inflammatory responses.

Microglia and astrocytes are the first to react after ICH [38]. The hematoma comprises diverse constituents, such as RBCs and detrimental RBC lysates. Remarkably, these constituents can activate microglia through distinct pathways. Recent studies indicate that culturing microglia with CD36 leads to their activation and upregulation of proinflammatory gene expression [39]. The breakdown of RBCs triggers microglial activation through TLR4 [40, 41], induced by hemoglobin and heme. The process modulates the gene expression of proinflammatory signaling factors and involves the transmission of the NF‐κB signaling pathway. Subsequently, the inflammatory immune response engages the NF‐κB signaling pathway, resulting in vasogenic edema and impairment of the BBB [42].

Microglia play an important role in the whole process of secondary injury from the current research [43], and the free conversion of its M1 and M2 phenotypes is also the focus of research [44]. The main function of resident microglia activation is to clear the hematoma, but too much microglia activation will break the balance and cause more neuroinflammation, releasing factors such as IL‐1β, chemokines, ROS, and so on [45]. M1 phenotype microglia cells significantly increase during ICH. The studies indicated that the levels of proinflammatory cytokines, including IL‐1β, TNF‐a, and IL‐6, rise within 6 h after hemorrhage [46]. M2 phenotype microglia cells assist in the clearance of hematoma after ICH and promote healing by clearing cellular debris [47]. However, in the acute phase of ICH, M2 phenotype microglia cells may transform to M1, exacerbating neuroinflammation. Hence, regulating the phenotypic changes for microglia cells through intervention to inhibit inflammation is promising therapeutic approach for alleviating brain injury and edema after ICH [48].

#### 2.2.2 Blood–Brain Barrier Disruption

A sign of the secondary injury phase is disruption of the BBB, leading to vasogenic edema and extravasation of plasma proteins into the brain parenchyma. CXCL2 in natural killer cells (NK cells) is upregulated after ICH, which enhances the recruitment of neutrophils and aggravates the damage of BBB [49]. Inhibition of NK cells reduce brain endothelial cells (BECs) injury in BECs, while the expression of tight junction proteins (TJPs) is enhanced.

When blood flows into brain tissue, the coagulation cascade will be activated. Thrombin is an important component of the cascade reaction, the activated thrombin is an effective activator for BBB breakdown and inflammation [50]. It also induces the expression of chemokines and adhesion molecules. Thrombin primarily activates astrocytes and microglia further through protease‐activated receptors. Microglia are activated by different signaling factors, mediated by CD36 in RBCs, thrombin reactions, and heme through the TLR4 signaling pathway [51]. This leads to excessive activation, resulting in the production of more ROS [52], TNF‐α, and cytokines, exacerbating neuroinflammation. Simultaneously, the recruitment of these inflammatory cells further damages the BBB. Additionally, current research suggests that thrombin has neuroprotective effects at very low concentrations.

The accumulation of activated microglia/macrophages at the site of injury will directly affect the permeability of the BBB [53], leading to inflammation and neuronal death by releasing proinflammatory factors, and changing the permeability of the BBB by activating BECs through the expression of the chemokine CCL2. In addition, astrocytes and microglia will induce the expression of matrix metalloproteinase‐9 (MMP‐9) [54]. The expression of MMP is related to the subsequent changes in brain edema and the degree of damage to the BBB [55]. MMP induces BBB destruction by degrading endothelial basement membranes and TJPs. Studies have shown that the reducing the expression of MMP‐9 and MMP‐12 can alleviate edema, the aquaporin (AQP) have found that its expression increases after acute brain injury and is related to angiogenic edema. Reducing the expression of AQP can alleviate brain edema and stabilize BBB [56]. The specific mechanism has not been clarified yet and it may also be a potential therapeutic target.

#### 2.2.3 Excitotoxicity Network

The continuous disruption of the BBB and brain tissue damage promotes the excessive release of glutamate [57], and excessive glutamate accumulation sustains the activation of NMDA receptors, AMPA receptors, and metabotropic glutamate receptors [58]. NMDA receptors are considered to play an important role in the formation of excitotoxicity and induce Ca^2+^ influx and intracellular signal cascade reactions [59]. Continuous stimulation of glutamate receptors can lead to excitotoxicity, thereby causing neuronal damage [60]. Under normal conditions, astrocytes can effectively uptake and maintain brain homeostasis [61]. However, extracellular glutamate levels are elevated in astrocyte dysfunction after ICH. And activated microglial cells secrete the proinflammatory factors IL‐1b and TNF‐α, which further promote the release of glutamate [62], therefore exacerbating neurotoxicity. In addition, oxidative stress caused by iron accumulation in ICH further exacerbates excitotoxicity [63]. Therapeutic strategies have been developed for neuronal death caused by neurotoxicity, such as the use of NMDA receptor antagonists and the enhancement of glutamate transporter proteins [64], and eliminating the increase in glutamate levels by regulating the activity of astrocytes has also provided an important direction for alleviating excitotoxicity.

### 2.3 Chronic Remodeling Phase

The chronic remodeling phase occurs after primary and secondary injuries. This phase lasts for months or even years. Although it is a recovery phase, it often leads to adverse reactions, the tissue structure and neural connectivity are changed in the brain. The brain's own protective mechanism promotes the beginning of endogenous neural repair, but due to excessive activation of glial cells, glial scar formation and failure of functional neural circuit reconstruction led to persistent neurological dysfunction in patients. Understanding these mechanisms will help develop targeted therapeutic interventions to promote neurological recovery.

#### 2.3.1 Gliotic Scar Formation

Glial scar formation represents a major impediment to neural repair after ICH. And studies have shown that the function of microglia and astrocytes changes during different periods of ICH [65]. Microglia in a state of persistent activation during the chronic phase are generally regarded as harmful and will form dense glial scars around the injury site, which is beneficial to neuroinflammation but can form physical barriers, hindering the regeneration of nerve axons and the occurrence of neural plasticity [66]. Eliminating neurotoxic microglia in the chronic phase can significantly improve neurodegeneration. Zheng's research indicated that in the chronic stage of ICH, microglia may interact with astrocytes through IGF1/IGF1R ligand receptors, and microglia‐derived IGF1 induces astrocyte scar formation by activating the mTOR signal [65]. Neurotoxic astrocytes occupy a major component of the cellular scar, thereby impeding nerve repair. Similarly, in other central nervous system diseases such as the neural repair process of spinal cord injury, astrocytes play a beneficial role in the acute stage, but show obstacles to neural recovery in the chronic stage. Recent studies have shown that regulatory T cells (Treg) are believed to be able to reduce excessive immune responses, inhibit excessive proliferation of astrocytes and thereby promote the recovery of neurological function after stroke [67]. Targeted regulation of astrocytes may be the key to enhancing neural repair.

#### 2.3.2 Failed Circuit Reconstruction

Stroke can lead to neuroinflammation, promote neuronal damage, cause neural circuit interruption, and aggravate the patient's neurological dysfunction [68]. Studies have shown that at the cellular level, axonal germination begins in the chronic phase and attempts are made to reconstruct neural circuits, which are continuously recover through the formation of synapses and the growth of dendrites [69]. It is worth noting that the neurogenesis effect after ICH is limited and does not meaningfully contribute to the reconstruction of brain circuits [70]. The spontaneous NSCs will multiply and migrate to the lesion area during the chronic stage of ICH [71]. However, due to the poor microenvironment after ICH, which is not conducive to endogenous neurogenesis, the majority of the cells most undergo apoptosis in the lesion area. Ultimately less than 5% of the cells are able to function, which is not sufficient to restore cerebral circuits. The failure of neural circuit reconstruction emphasizes the necessity of promoting neural repair therapy.

Rehabilitation interventions have been widely used to improve motor dysfunction after stroke, studies have demonstrated that rehabilitation training within 2 weeks can have a positive impact on synaptic reorganization and connection [72]. In addition to conventional rehabilitation, emerging brain–computer interface (BCI) techniques can be used in conjunction with conventional rehabilitation to achieve better recovery [73], but many stroke patients still experience long‐term neurological deficits.

In summary, the damage caused by ICH due to immunology activation, and the release of toxic substances, these factors lead to primary and secondary damage to neural cells. Nevertheless, besides explaining the mechanism of injury, the timing of intervention is also important. Considering the dynamic and rapid changes in the pathological process of ICH, identification of the most effective time window of treatment is extremely necessary for achieving utmost neuroprotection along with functional recovery.

## 3 Therapeutic Windows: Temporal Precision in Intervention

**FIGURE 3:**
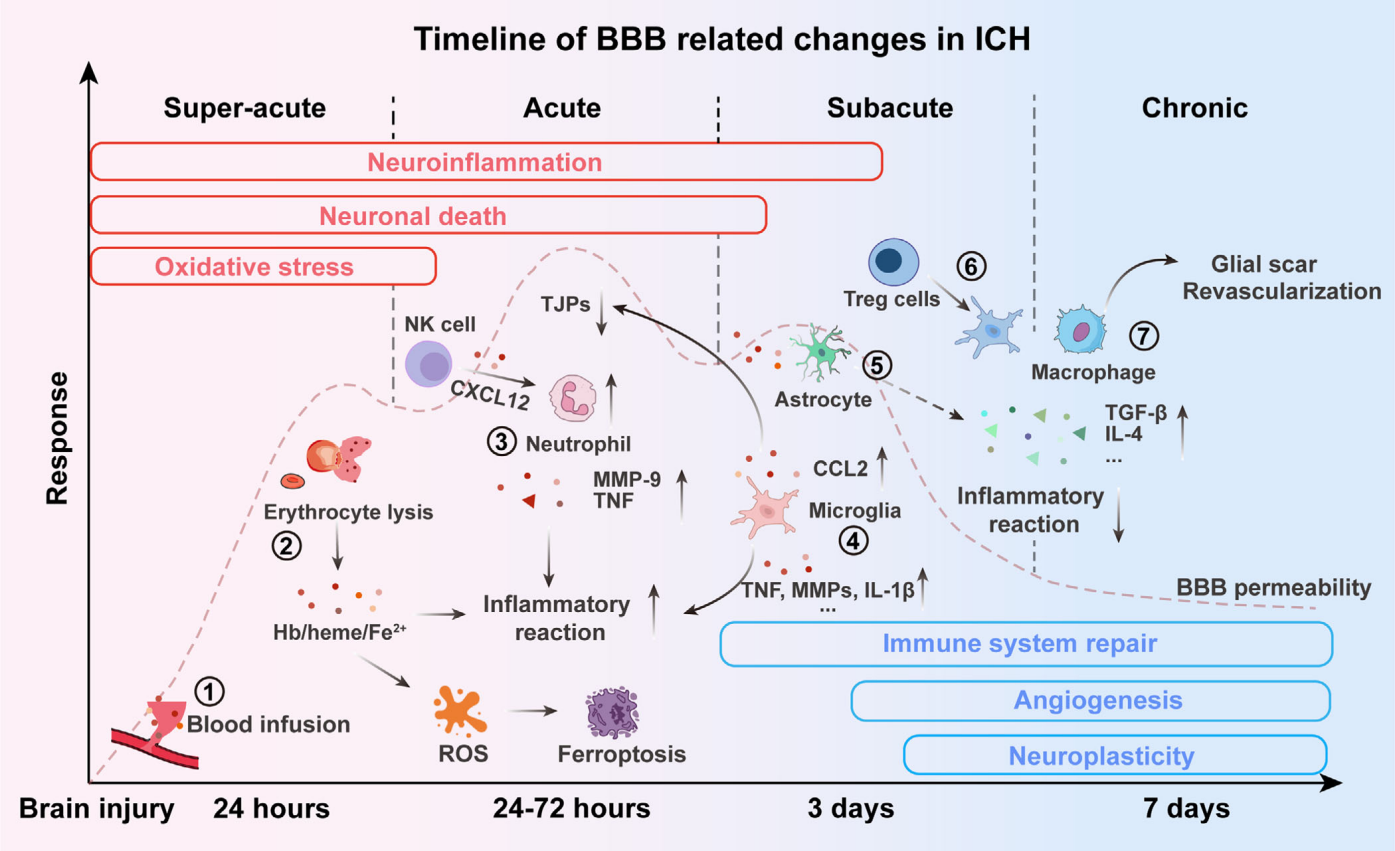
Timeline of BBB‐related changes in ICH. Blood vessel rupture results in the entry of blood into the brain parenchyma, leading to the formation of a hematoma that compresses the BBB (1). The lysis of red blood cells releases hemoglobin and iron, which trigger the Fenton reaction, generating reactive oxygen species that damage vascular endothelial cells (2). Oxidized heme and iron are critical to the breakdown of the BBB. Furthermore, the extravasation of blood components, including thrombin and degradation products of red blood cells, initiates an immune inflammatory response surrounding the hematoma. NK cells produce CXCL12 to enhance neutrophil aggregation (3), while activated astrocytes and microglia release inflammatory factors such as TNF and IL‐1β, downregulating TJPs and compromising the integrity of the BBB (4). The inflammatory response and activation of MMPs significantly contribute to the destruction of the BBB. Subsequently, endogenous repair mechanisms are gradually activated, immune cells release anti‐inflammatory factors that reduce the expression of IL‐1β and TNF (5), and Treg cells inhibit the inflammatory response by modulating the excessive activation of microglia/macrophages, thereby decreasing the formation of excessive glial scars (6). Concurrently, microglia/macrophages express growth factors associated with inflammatory recovery to facilitate the repair of the BBB (7).

The pathophysiological response of ICH changes over time, requiring a staged treatment strategy. Developing precise treatment plans for different stages (hyperacute, subacute, and chronic) can not only improve the therapeutic effect, but also minimize off‐target effects and toxicity. Providing appropriate treatment at each stage is essential to improve the survival rate and prognosis of ICH patients [8]. This section delineates current and emerging therapeutic modalities across these critical windows (Figure 3).

### 3.1 Hyperacute Phase (<6 h)

The hyperacute phase is a series of pathophysiological responses occurring within 6 h of ICH, characterized by vascular rupture and bleeding, rapid hematoma expansion, and initial inflammation. Most patients with ICH will experience hematoma expansion, the main intervention currently aims to stabilize the hematoma and reduce mechanical damage, but the urgency of neuroprotection must also be recognized.

#### 3.1.1 Hematoma Evacuation vs. Neuroprotection Dilemma

ICH is a neurological emergency, and whether to prioritize the management of the hematoma or opt for neuroprotection in the hyperacute phase remains a clinical dilemma [74]. The hyperacute phase of ICH is primary brain injury. And the purpose of early surgery is mainly to reduce the mass effect of hematoma on surrounding tissues and prevent the toxic components released by blood from further causing secondary damage [75]. For early hemorrhages that are large or significantly affect the midline, which can improve survival, surgical removal of the hematoma is used only as a life saver [76]. However, for most cases of ICH on the surface of the brain, clinical data statistics from the International Surgical Trial of Intracerebral Hemorrhage showed that the effectiveness of early surgery was not obvious [77]. If subsequent neurological recovery is not taken into account, the use of minimally invasive surgery might be reasonable over conventional craniotomy, minimizing additional trauma to uninjured brain tissue and resulting in a lower incidence of surgical complications compared with hematoma removal. However, the timing of its choice of minimally invasive is still unresolved, and conclusions on its subsequent neurological recovery are still lacking relevant research support.

Along with surgical considerations, early neuroprotection must be implemented during this critical window. Experiments have shown that the further expansion of hematoma can be inhibited by forcibly lowering blood pressure with continuous use of antihypertensive drugs [78], and the finding that the risk of neurological deterioration is lower when the patient's systolic blood pressure is less than 140 mmHg suggests that antihypertensive therapy is beneficial and safe in the acute phase [79]. The method to prevent the further expansion of hematoma is to use drugs that alter the coagulation cascade reaction, and use of vitamin K antagonists is associated with the expansion of hematoma and subsequent prognosis [80]. The recent phase II clinical trial results of recombinant factor VIIa indicate that treatment during the acute phase can limit the expansion of hematoma and improve treatment outcomes [81, 82].

The key challenge is to synchronize external surgical intervention with neuroprotection. Although surgery can address the occupying effect of the hematoma, it may further exacerbate the secondary injury, and neuroprotection with medication alone may be ineffective for large hematomas. Further investigation of bioimaging tools to detect certain markers that indicate the rate of subsequent hematoma growth before deciding on treatment may also be a focus of future research.

#### 3.1.2 Iron Chelation Strategies

Iron is essential for normal brain metabolism and therefore iron dysregulation impairs brain function [83]. RBC lysis was observed after ICH followed by release of large amounts of neurotoxic iron into the brain parenchyma leading to neurotoxicity and brain cell death [84]. Iron toxicity becomes evident within hours of hemorrhage onset, making the hyperacute phase optimal for iron chelation interventions.

Deferoxamine (DFO) is an iron‐chelating agent that penetrate the BBB to form a stable complex with free iron, inhibiting further free radical Fenton reactions and alleviating cell death, indicating that it may be available for future ICH treatment [85]. In the angiotensin II‐induced hypertension mouse model of ICH, injection of DFO can effectively alleviate iron overload and inhibit secondary hemorrhage, and there have been a number of clinical trials evaluating the effect of DFO in the treatment of ICH [86], and the results showed that low‐dose DFO was well tolerated in patients with no serious adverse effects, which preliminarily proves the clinical safety of DFO [87]. Unfortunately, other studies showed that low‐dose DFO may not make an exciting difference in neurologic recovery outcomes after ICH [88]. And the high dose of DFO has been discontinued because of serious adverse effects [89], so in order to further improve the BBB penetration rate and prolong the half‐life of DFO, liposomes loaded with DFO have been a promising targeted delivery system, which can minimize systemic toxicity by targeting the controlled release from the hematoma, and a study has further demonstrated the ability of liposomes to reduce the production of ROS compared with free DFO to improve the neurological prognosis. Clinical studies are needed to further evaluate the safety and efficacy of liposome DFO.

Based on the proposed iron chelation mechanism, more and more drugs with affinity for iron have been proposed and studied [90]. The clinic hopes that the mechanism of action of chelating ferrous iron or iron can be exerted to further improve the prognosis of patients with ICH. Although there is a large amount of literature on iron toxicity, there are still many differences and problems that need to be solved. The permeability and bioavailability of BBB are also the main factors limiting the therapeutic effect of ICH. Recent studies have also been conducted to expand the effect of neuroprotection by selecting emerging drug delivery strategies [91]. The development of multiple therapeutic strategies also demonstrates the current clinical need for a breakthrough approach to the treatment of ICH.

### 3.2 Subacute Phase (7 days–1 month)

Subacute phase is the next phase of acute phase, which lasts about 1 month and is characterized by secondary mechanisms of injury, neuroinflammation, and vascular damage that aggravate the death of nerve cells, but at the same time the immune system will begin to undergo gradual repair during this phase, which provides a therapeutic window to modulate the inflammatory response to promote repair and regeneration.

#### 3.2.1 Anti‐inflammatory Reprogramming

The continuous activation of microglia and the continuous release of cytokines by macrophages aggravate neuroinflammation further lead to the destruction of the BBB, and peripheral edema caused by hematoma leads to neuronal apoptosis. It can be considered that this stage may play a crucial role in the subsequent recovery of neurological function. With the continuous deepening of research, it is found that an active immune system response for repair while neurotoxicity occurs. In the acute phase, microglia are mainly M1 phenotypes that expand the inflammatory response, and in the subsequent subacute phase, the phenotype gradually changes to the M2 anti‐inflammatory phenotype [92]. This discovery makes the research to consider transforming their phenotype to promote tissue repair.

Anti‐inflammatory reprogramming has received extensive attention. Minocycline is a tetracycline derivative that can exert anti‐inflammatory effects by inhibiting M1 polarization and is considered a potential drug for the treatment of ICH [93]. The study has been demonstrated that minocycline is able to reduce periprocedural edema and inhibit cell death, clinical research on minocycline for ICH patients is also underway, and the preliminary results have demonstrated an acceptable safety profile, but effectiveness still needs to be confirmed [94]. In addition, current studies have also shown that nuclear receptor agonists targeting peroxisome proliferator activating receptors (PPARs), such as rosiglitazone and pioglitazone, can inhibit proinflammatory factors while activating microglia and macrophages to promote inflammation absorption, the mechanism of action is still under further study [95].

Targets or markers related to neurological diseases have also attracted attention. Studies have shown that in ICH model established by self‐extraction of blood, Homer1 protein was injected into the hemorrhage site and found to be able to improve the prognosis by inhibiting the transformation of glial cell phenotype through the MAPK signaling pathway [96]. Cell treatments, including mesenchymal stem cells (MSCs) therapy and exosome‐based therapy, have shown particular promise during this window [97]. Combining it with the exosomes derived from MSCs, the pharmacokinetics of Homer1 can be significantly improved, and the protein can be more easily targeted to the site of injury to promote the transformation of the anti‐inflammatory phenotype [98]. Emerging biomedical technologies provide more possibilities for expanding the therapeutic effect of ICH.

#### 3.2.2 Angiogenesis–Axogenesis

Promoting the progress of reconstructing the neural network in the subacute phase is crucial for the recovery of neurological function in the later phase. Therefore, actively promoting angiogenesis and facilitating neuronal plasticity is the key process to carry out endogenous self‐repair, and current studies have shown that VEGF can significantly promote the proliferation of vascular endothelial cells and the formation of vascular network [99]. In addition, exogenous injection of leptin has been shown to significantly reduce pericyte loss in chronic phase of ICH and significantly improve neurobehavioral function, suggesting that the formation of vascular networks can indeed improve the prognosis of ICH and provide scaffolding for neural axon growth [100].

However, in the acute phase of ICH, angiogenesis will further deepen the disruption of the BBB, and only in the subacute phase can it play a positive neurorestorative effect [101]. The difference in the role of different time stages also indicates the importance of corresponding treatment for ICH [102]. Angiogenesis‐promoting therapy should be started after 7 days, when the acute hemorrhage subsides. Moreover, excessive administration will also aggravate edema, so the application of intelligent drug delivery systems in the biomedical field has received clinical attention. Compared with direct in situ injection, the programmed release of VEGF based on hydrogels between 7 and 21 days can enhance the formation of vascular networks and provide better living space for axon growth in time, and achieve better prognosis [103]. Hydrogel scaffolds with continuous release of VEGF and BDNF also provide a promising approach for subacute intervention [104].

### 3.3 Chronic Phase (>1 month)

Increasing evidence suggests that the chronic phase plays an important role in subsequent neural circuit remodeling and functional recovery. Oxidative stress and cell death caused by early ICH seriously threaten the subsequent neurological function recovery. Although the immune system will carry out self‐repair of neurons, the effect is still minimal. And the formation of neural scars and the failure of neural circuits limit the occurrence of neural recovery. Intervention during this period effectively improves neuronal plasticity and promotes successful recovery of cerebral neural loops, suggesting a favorable prognosis.

#### 3.3.1 Circuit Remapping Enhancers

The reorganization and complete repair of neural circuits may last for several months. Compared with the previous stage, the chronic stage provides a longer window of opportunity, which interventions to the neural circuits can provide a tremendous help in the recovery of neurological function after stroke. This opportunity has stimulated interest in therapies specifically designed to enhance neural circuit remapping.

Neurotrophic factors play an important role in neural remodeling in brain diseases, promoting dendritic remodeling and synaptic strengthening [105]. 7,8‐Dihydroxyflavone, a small‐molecule BDNF mimetic, has been recently shown to have a protective effect on the brain and neuronal cells against toxin‐induced damage, and administration in the chronic phase shows the potential to enhance neurogenesis [106]. In addition, MSCs overexpressing glial cell‐derived neurotrophic factor (GDNF) in treatment of rats with ICH can inhibit neuroinflammation, promote the survival of neurons, and enhance the plasticity of neural synapses, which has attracted further research attention [107].

Noninvasive brain stimulation techniques include transcranial magnetic stimulation (TMS) and direct current stimulation, both of which promote the occurrence of neuroplasticity by regulating cortical excitability [108]. Study has showed that continuous 5 days application of TMS in collagenase‐induced ICH mice significantly reduced cerebral edema and improved neurological function [109]. And noninvasive neurostimulation therapy has been tested in clinical for poststroke dysphagia, and statistical analysis indicated 27 intervention groups had significant improvement in dysphagia symptoms [110].

Based on the basic electrical stimulation, BCI technology promoting neural recovery represents the most promising new approach in modern rehabilitation [111]. BCI technology have enabled external closed‐loop systems to effectively detect endogenous patterns of neural activity signals, thus providing targeted stimulation to promote the complete mapping of brain circuits and enhance the ability of patients in performing some basic daily activities [112]. However, the symptoms of depression after ICH are common, this technology may be able to improve the emotional and cognitive rehabilitation of patients through neurofeedback training, and studies have concluded that motor, sensory, and cognitive rehabilitation will have a synergistic effect on the prognosis of ICH [113], so the multimodal and multisignal BCI technology is also an important direction of the research, and the progress of the current research on BCI technology will also be discussed in the next section.

The complex pathophysiological cascade after ICH presents a significant challenge to the development of effective therapeutic interventions. Although current clinical treatments focus on hematoma evacuation, blood pressure control, and rehabilitation, these strategies are limited in their capacity to address the underlying cellular damage and to promote functional recovery. Based on the multiple injury mechanisms outlined above, there is an urgent need for comprehensive strategies for nerve regeneration. A growing number of review articles have discussed various therapeutic approaches for ICH (Table 1). However, the scope, focus, and proposed strategies vary considerably across studies, and a comprehensive discussion of emerging strategies and clinical translation is needed further.

**TABLE 1:** Summary of recent review articles on the treatment of ICH.

Title	Scope	Year	References
Novel targets, treatments, and advanced models for intracerebral hemorrhage	This review summarizes the current therapeutic targets and emerging treatment methods for ICH.	2022	Zille et al. [114]
Small interfering RNAs based therapies for intracerebral hemorrhage: challenges and progress in drug delivery systems	This review introduces the latest methods of small interfering RNA delivery for the treatment of ICH and discusses the research status of nanotechnology in the treatment of ICH.	2022	Almarghalani et al. [115]
The development of drug delivery systems for efficient intracranial hemorrhage therapy	This review describes a variety of drug delivery systems for the treatment of ICH.	2023	Yu et al. [116]
Application of nanomaterials in the treatment of intracerebral hemorrhage	This review summarizes the research progress of nanomaterials in the treatment of ICH based on clear pathological mechanisms.	2023	Zhang et al. [117]
Injectable hydrogels in central nervous system: unique and novel platforms for promoting extracellular matrix remodeling and tissue engineering	This review introduces that injectable hydrogel can mimic the characteristics of brain tissue and help to improve neural stem cell differentiation.	2023	Hasanzadeh et al. [118]
Endovascular brain–computer interfaces in poststroke paralysis	This review summarizes the research status of the role of BCI technology in neural recovery.	2024	Brannigan et al. [119]
Intracerebral hemorrhage‐mechanisms, diagnosis and prospects for treatment and prevention	This review introduces new drugs that are being studied for the treatment of ICH.	2024	Thomas et al. [120]
Novel therapeutic mechanisms and strategies for intracerebral hemorrhage: focusing on exosomes	This review introduces the therapeutic potential of exosomes in ICH and some miRNA‐mediated molecular biological mechanisms.	2024	Jiang et al. [121]
Evolving therapeutic landscape of intracerebral hemorrhage: emerging cutting‐edge Advancements in surgical robots, regenerative medicine, and neurorehabilitation techniques	This review summarizes innovative therapies including robot‐assisted minimally invasive surgery, stem cell transplantation, in situ neuronal reprogramming, and BCI.	2025	Chen et al. [122]
Nanomaterial technologies for precision diagnosis and treatment of brain hemorrhage	This review discusses the progress in nanomaterials for diagnosis and therapy.	2025	Zhang et al. [123]

## 4 Regenerative Engineering: Converging Biology and Technology

Hemorrhage into brain parenchyma leads to the formation of hematoma, leading not only to primary mechanical injury but also secondary injury caused by inflammatory cascades, oxidative stress, and neuronal death. Although surgical treatment for the hematoma has improved survival, effective therapeutic options for promoting neurological recovery remain limited [124]. So ICH is still the largest cause of neurological dysfunction and even death around the world. In recent years, regenerative engineering has become a promising approach, it combines advanced materials science, stem cell biology, and biomedical engineering, ultimately enhancing functional recovery in patients by promoting targeted neural regeneration and circuit repair [125].

### 4.1 Exogenous Cell Therapy

Stem cells have low differentiation properties, which enable them to differentiate into a variety of cell types [126]. Whether in vivo and in vitro, they can regulate proliferation and differentiation rates to adapt to the demands of the surrounding environment, thereby ensuring the homeostasis of tissues and organs. Taking advantage of these characteristics, stem cell transplantation has become a promising method for treating ICH [127]. Studies have shown that diverse types of stem cells can reduce brain damage and improve functional outcomes after stroke through multiple mechanisms [128].

#### 4.1.1 MSC‐Derived Therapeutics

MSCs are considered as a novel strategy for treating ICH due to their ability to reduce inflammation, promote angiogenesis, and enhance neurogenesis [129]. Treatment of ICH rats with MSCs can reduce apoptosis and enhance neuroprotection [130]. Hypoxic preconditioning method can increase the survival rate of stem cells in the brain tissue. The studies show that this method can delay cell aging through the PI3K signaling pathway and reduce the apoptosis of microglia after ICH.

Various pretreatment methods have all achieved better experiment results, and more and more methods are improving the efficacy of transplanted stem cells. To alleviate secondary damage caused by oxidative stress, Huang's et al. proposed using curcumin to pretreat olfactory mucosa‐derived MSCs [131]. In vivo and in vitro experiments were conducted, and the results consistently demonstrated that the pretreated MSCs exhibited enhanced antioxidant capabilities and mitigated neuronal damage caused by ferroptosis. Consequently, this preconditioning method is regarded as a novel therapeutic strategy for stem cell transplantation.

**FIGURE 4:**
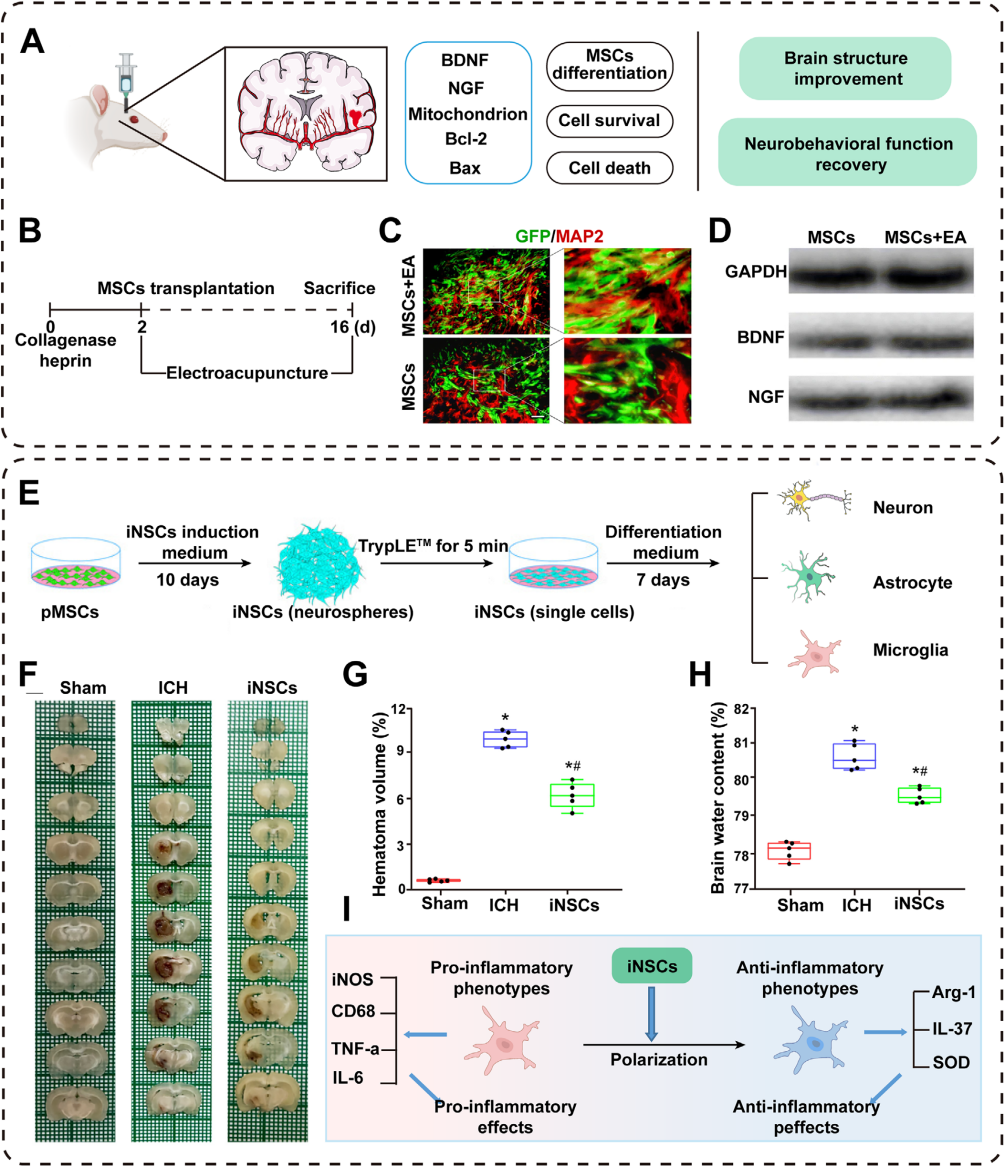
Cell transplantation promotes neural recovery. (A) Combined therapy can promote the differentiation of MSCs and cell survival, thereby improving the rehabilitation of neurological function [132]. Copyright 2022, Springer Nature. (B) Timeline showing treatment options for EA stimulation and/or MSC transplantation after ICH. (C) The MSCs + EA group significantly increased the expression neuronal markers (MAP2). (D) WB showed the expression levels of BDNF and NGF around the hematoma on the 14th day. (E) Inducing human placental mesenchymal stem cells (pMSCs) to differentiate into NSCs [144]. Copyright 2023, Cell Press. (F) Hematoma size in each group on day 7 of ICH. (G) Quantitative analysis of hematoma volume in each group. (H) Quantitative analysis of water content in the injured side of brain tissue in each group on the 7th day of ICH. (I) iNSCs modulate microglia polarization.

In addition to pretreatment of stem cells, the combined therapeutic strategies are also considered to be beneficial for enhancing treatment effects after stem cell transplantation. Deng et al. [132] investigated the potential of electroacupuncture (EA) as an adjunctive therapy to improve the efficacy of MSC transplantation in ICH rats (Figure 4A,B). Compared with the transplantation of MSCs alone, combining the application of EA significantly increased the expression of neuronal markers (MAPs) and promoted the differentiation of MSC cells into neurons (Figure 4C). After transplanting MSCs into ICH rats, EA stimulation was applied, revealing that EA facilitated the neural differentiation of stem cells and enhanced neurological function (Figure 4D). The combined therapy may augment posthemorrhagic neurological recovery through various pathways.

Moreover, the research trends over the past 5 years indicated that the beneficial therapeutic effects of MSCs are closely related to their paracrine properties [133]. Their extracellular vesicles (EVs) have become the focus of research. Exosomes usually contain many specific proteins and can cross the BBB to exert anti‐inflammatory and neuroprotective effects, leading to the study of the therapeutic effects of exosomes [134].

The microRNA present in MSC‐EVs has been identified as the key substances for therapeutic effect. Exosomes enriched with miR‐146a‐5p derived from bone marrow MSCs (BMSCs) alleviate ICH by inhibiting neuronal apoptosis and M1 polarization of microglia [135]. Hu et al. [136] demonstrated that BMSC‐EVs enriched with miR‐23b could alleviate oxidative stress and pyroptosis after ICH. BMSC‐EVs have been found to alleviate neuroinflammation after diabetic ICH through the miR‐183‐5p/PDCD4/NLRP3 pathway [137]. Sun et al. [138] revealed that BMSC‐EVs enriched with miR‐150‐3p can reduce cell apoptosis and inflammatory factors by modulating the TRAF6/NF‐κB signaling pathway. Furthermore, this study provides the initial evidence that MSC‐EVs can influence gut microbiota metabolism, thereby enhancing neurological function recovery after ICH. Furthermore, previous research has shown that miR‐100‐5p in the exosomes of human umbilical cord MSCs has the ability to alleviate cellular inflammation [139]. Nan et al. [140] investigated the therapeutic potential of these exosomes, revealing their ability to downregulate target factors through the TLR4/NF‐κB signaling pathway, enhancing neurological function recovery, thereby providing scientific evidence supporting their clinical applications. Using MSC‐EVs therapy has shown significant potential [141]. However, the clinical translation of this method is limited by current limitations of exosome isolation techniques, which include strict equipment requirements, time‐consuming procedures, and complex identification processes. More experimental evidence is required to determine the safety and effectiveness of this method.

#### 4.1.2 Cell Programming Precision Approaches

Direct neuronal reprogramming without reverting to pluripotency has become an effective approach for generating specific neuronal subtypes [142]. It has been proven that *achaete‐scute complex‐like 1* can be used to reprogram glial cells into induced neurons, which holds the potential to assist patients in rebuilding their brain circuits [143]. This approach reduces tumorigenic risks associated with pluripotent intermediates while maintaining the advantages of autologous sourcing. As previously mentioned, MSCs exhibit therapeutic effects in ICH, inducing MSCs to differentiate into NSCs (iNSCs) in vitro for transplantation offers a more targeted and effective treatment for neural injury (Figure 4E) [144]. Cell reprogramming gives them the potential to differentiate into neural cells [145]. The results of the study showed that the hematoma volume and brain water content in the iNSCs group were significantly reduced 7 days after ICH (Figure 4F–H). Behavioral test results showed that iNSCs transplantation can better improve neurological deficits. Most importantly, the results showed that iNSCs transplantation can play an anti‐inflammatory role by regulating the phenotype of microglia (Figure 4I). Many cutting‐edge studies have indicated that regulating the phenotype of microglia may be a promising therapeutic approach.

Compared with conventional neural progenitor cells (NPCs), iPSCs‐derived NPCs with region‐specific identities have better integration capabilities. iPSCs have enhanced immunomodulatory properties [146]. iPSCs treatment improves the recovery of neurological dysfunction in experimental ICH models, and induced pluripotent stem cell‐derived non‐neuronal support cells have shown significant therapeutic effects [147]. Mature astrocytes can be generated by *SOX9* overexpression for 6 days for the treatment of brain diseases [148]. The findings suggested that inducing NSCs could be a promising treatment approach for ICH and other neuroinflammatory diseases.

The emergence of the gene editing technology CRISPR has made it possible to achieve more precise reprogramming of stem cells to enhance neural regeneration through the activation or inactivation of specific genes [149]. Additionally, utilizing gene transduction technology, NSCs can be modified to express particular growth factors or neuroprotective factors prior to transplantation, thereby boosting their therapeutic efficacy [150]. Wakai et al. [151] isolated NSCs from transgenic mice overexpressing *SOD‐1* and applied them in ICH mouse model induced by autologous blood. On the 5th and 14th days after the experiment, tissue samples were collected for analysis. It was found that the increased expression of *SOD‐1* had an antioxidant effect, protecting the adjacent tissues of the lesion, thereby enhancing the survival rate of NSCs transplantation. GDNF has been shown to play an important role in neuroprotection after ICH, to explore the potential of overexpressing GDNF (MSCs/GDNF) by gene editing, Jiang et al. [107] conducted experiments by transplanting MSCs/GDNF into ICH rats. The findings indicated that MSCs/GDNF can enhance neuronal survival and improve the neurobehavioral function in treating rats following ICH. The impact of oncogene *ski* overexpression on neural recovery was investigated by Zhai's team [152]. Mice were injected with adenovirus‐mediated overexpression genes to induce a brain injury mice model. The findings ultimately suggested that *ski* overexpression could augment neural behavioral recovery and facilitate neuronal regeneration and maturation.

The advent of gene editing technology has significantly advanced the treatment of ICH [153]. However, there are still several limitations to this technology. For example, the off‐target problem associated with gene editing needs to be solved [154]. Furthermore, there are clinical demands for enhanced biological safety, and the potential side effects of heightened gene expression require further verification [155].

### 4.2 Endogenous Stem Cell Modulation

Although exogenous stem cell transplantation provides direct cellular and paracrine protection for injured brain tissue, regulating endogenous stem cells to promote brain nerve regeneration has also attracted extensive research. Enhancing the generation of NSCs represents a potential treatment approach for neurological disorders including ICH [156]. Research on stroke models has demonstrated that endogenous NSCs can migrate to the site of cerebral infarction. ICH leads to the proliferation and differentiation of endogenous NSCs in the subventricular zone of the mammalian brain, and the newly formed neurons can migrate to the damaged brain area to replace dead neurons [157]. This indicates the significant role of NSCs in promoting recovery of the nervous system after stroke. Therefore, stroke treatments based on NSCs have received considerable attention.

#### 4.2.1 NSC Niche Reactivation

Precisely regulating the proliferation and differentiation of NSCs is essential for generating a specific number of neurons in the brain [158]. After ICH, NSCs will respond to the need for repair by proliferating, migrating to the injured area, and differentiating into new neurons. Recent research suggests that targeting the regulatory mechanisms activated by NSCs post‐ICH can enhance endogenous neurogenesis, inhibit apoptosis, and enable NSCs to actively migrate to the site of brain injury. This contributes to the repair of neural damage after ICH [159].

The delivery of growth factor is an important approach for NSC niche stimulation. The Wang's team used recombinant human insulin‐like growth factor in ICH mice by transnasal administration, and demonstrated its ability to inhibit inflammatory polarization of microglia and promote endogenous nerve regeneration through IGF‐1R stimulation and the TLR4/NF‐κB pathway [160]. NGF and BDNF also play important roles in neural development. A clinical experiment (ChiCTR1800020258) proved that treating patients with mNGF could significantly improve their neurological function [161], and Sun et al. [162] also found applying acupuncture could increase the expression of BDNF and protect nerve cells. The authors further found that urokinase‐type plasminogen activator (uPA) could enhance the neuroprotective effect of BDNF in ICH rats; BDNF in combination with uPA could significantly stimulate neuroprotection and brain recovery after stroke [163]. Similarly, bFGF was also demonstrated to increases NSC proliferation and have a protective effect on neuronal cells [164], and various growth factors play a significant role in promoting neural regeneration after ICH.

#### 4.2.2 Epigenetic Reprogramming

Epigenetic mechanisms affect NSC fate and neural differentiation, the harsh microenvironment of ICH leads to aberrant epigenetic modifications that affect NSC function, and reprogramming epigenetic inheritance offers promising approaches for treating ICH. Histone modification is the main mechanism of epigenetic regulation of gene expression. Early studies have shown that nonspecific histone deacetylase inhibitors can reduce neural damage, and valproic acid administration can induce neuronal differentiation and mediate the expression of neuroplasticity genes [165]. Histone 3 lysine 9 trimethylation (H3K9me3) is a well‐studied and conserved epigenetic modification; Lan et al. [166] has been shown to participate in neuronal ferroptosis during ICH. When methyltransferase Suv39h1 or siRNA inhibitors are used to inhibit H3K9me3, it would exacerbate ferroptosis, providing new insights into the molecular mechanism of ferroptosis in ICH. Noncoding RNA represents an emerging epigenetic regulatory factor and has become a new candidate drug for the treatment of ICH. miR‐194‐5p has been reported to play an important role in brain and neurodegenerative diseases. Further studies have found that injecting miR‐194‐5p agomir can significantly reduce the nerve damage in rats and inhibit neuroinflammation, which provides new ideas for the treatment of ICH [167]. In addition, microRNA‐152 has also been confirmed by studies that overexpression in microglia can reduce heme‐induced inflammation and ROS production, thereby playing a protective role in neurons [167]. Moreover, the discovery and validation of novel microRNAs further suggests that targeting neural differentiation can further enhance endogenous neurogenesis [168], and further studies are needed in the future to identify specific epigenetic targets after ICH.

Exogenous stem cell transplantation and endogenous cell regulation represent promising strategies for enhancing self‐repair and neural regeneration after ICH [169]. However, their therapeutic efficacy is still constrained by factors such as an unfavorable microenvironment and inefficient cell activation [170, 171]. Additionally, transplant routes and timing will affect the therapeutic effect [172]. The clinical translation of stem cell transplantation remains challenging due to ethical considerations [173]. To further enhance nerve regeneration, many new biological materials have been used to improve the hematoma microenvironment. Promoting endogenous neurogenesis not only improves biocompatibility but also contributes to functional recovery after treatment [174].

### 4.3 Intelligent Biomaterial Platforms

As previously mentioned, promoting nerve regeneration is widely considered to have significant value in stroke treatment. Restoring the lost nerve cells due to injury can accelerate neural recovery. Subsequent studies have identified that the presence of endogenous NSCs can be activated after brain injury to replenish neurons lost due, but the complex pathological environment of the brain often hinders endogenous nerve repair [124]. To overcome these challenges and improve regenerative effect, the development of intelligent biomaterial platforms has attracted growing interest. These platforms provide a more beneficial microenvironment, enable targeted delivery, and respond intelligently to pathological signals, thereby synergizing with endogenous repair mechanisms [125].

#### 4.3.1 Hematoma‐Responsive Systems

The formation of hematoma and the release of blood components will trigger a series of pathophysiologic reactions. In response to this phenomenon, advanced biomaterials have been designed to provide appropriate treatment methods for promoting nerve repair. Engineered cell membrane coatings and nanoparticles targeting systems are both promising approaches [175]. There are some inherent limitations of cell therapy, to address this issue, recent studies have integrated nanotechnology, enabling cells to function better, improving the accuracy of treatment, and achieving better therapeutic effects.

The drug delivery system based on nanocarriers offers an innovative treatment approach for targeting the hematoma microenvironment. Nanocarrier systems also offer the potential to breach the BBB, which limits the therapeutic effect of drugs on ICH. Therefore, nanodelivery systems advances have demonstrated particular promise in targeting hematoma and regulating neuroinflammatory responses. Recently research indicates that certain anti‐inflammatory factors can regulate M1/M2 cell polarization, promoting hematoma resolution and functional recovery. But targeted delivery of anti‐inflammatory cytokines to hematomas remains challenging. Han's team devised a novel approach to this problem using nanoliposomes, comprising phosphatidylserine to fabricate nanoliposomes designed for the targeted delivery of IL‐10 [176]. Upon intranasal administration, functionalized nanoliposomes showed higher hematoma targeting efficiency, and compared with traditional delivery methods, significantly enhanced the immunomodulatory effect of IL‐10. Using of nanotechnology can cross the BBB and enhance drug stability. Abudurexiti et al. [177] designed a multifunctional solid lipid nanoparticle system that could simultaneously deliver curcumin and TGF‐β1 siRNA (siRNA/CUR@SLN) to the hematoma area. Their nanoparticle design achieved dual therapeutic effects: delivering curcumin to anti‐inflammatory effects, and targeting the regulation of TGF‐β1 expression. By intranasal administration, the targeting to the hematoma was further enhanced, effectively reducing neural inflammation and improving neurological prognosis. This conclusion emphasizes the great potential of nano‐liposome technology in treating ICH. The development of nanotechnology now offers various methods to integrate into nanomaterials, providing a platform for developing innovative therapies for ICH, thus further expanding the potential for future research.

**FIGURE 5:**
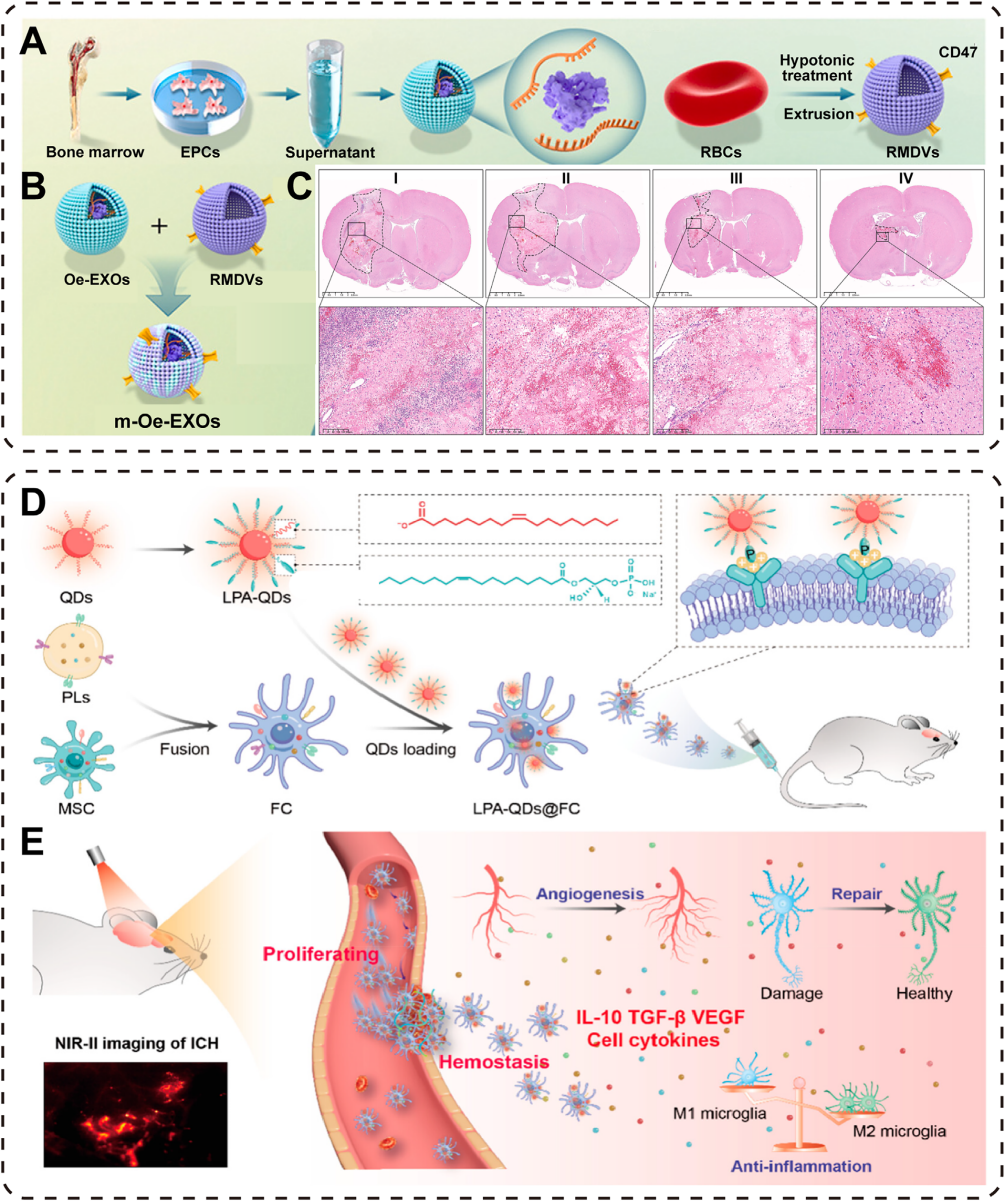
The functionalized cell membrane improves the treatment of nerve injury. (A) Isolation and extraction of EPC‐EXO from rat bone marrow. Extraction of CD47‐enriched RBC membrane‐derived vesicles (RMDVs) from RBCs. (B) The m‐Oe‐EXOs was prepared by mechanical coextrusion. (C) H&E staining image after ICH on the 3rd day, the dotted line is the hemorrhage area [179]. I: In situ injection of PBS, II: Intranasal delivery of PBS, III: In situ injection of m‐Oe‐EXOs, IV: Intranasal delivery of m‐Oe‐EXOs. Copyright 2025, Elsevier. (D) A visualized hybrid cell system was constructed by fusing MSC with platelets and loading lysophosphatidic acid (LPA)‐modified PbS quantum dots. (E) The treatment process can be visually tracked by NIR‐II fluorescence imaging [180]. Copyright 2023, American Chemical Society.

The functionalized cell membranes targeting hematoma can promote nerve recovery. The complex microenvironment of the hematoma hinders the effectiveness of exogenous therapies and the occurrence of endogenous neural repair after ICH [178]. In order to achieve targeted therapy, the design of functionalized cell membranes capable of responding to specific biological signals of the hematoma has become a hot topic in current research. Studies have shown that when ICH occurs, endothelial progenitor cells (EPCs) will be recruited to the damaged area for repair. Exosomes derived from EPCs (EPCs‐EXO) can target damaged blood vessels and promote nerve repair (Figure 5A) [179]. However, the repair capacity is limited due to the low proportion of exosomes reaching the target area. By encapsulating it with red blood cells rich in CD47, it has antiphagocytic properties and is strongly targeted to inflammatory areas (Figure 5B). The treatment effect is further improved compared with in situ injection by using the intranasal delivery method and significantly reduces damage around the hematoma (Figure 5C). In order to better increase the hematoma targeting and improve the retention of transplanted cells in the body, the strategy of fusion of cell membranes was first proposed by Wu's team [180]. Platelets were fused with MSCs using polyethylene glycol to form a hybrid cell. Furthermore, these fused cell membranes are loaded with PbS quantum dots modified with phospholipids, allowing for visualization of the ICH treatment process (Figure 5D). This offers a more intuitive understanding of the targeting and tropism of the hybrid cells at the hematoma site. The emergence of this approach indicated that by integrating the natural characteristics of different cells, achieving multifunctional ICH therapy has great potential. Additionally, the modification of functionalized cell membranes with quantum dots enables real‐time monitoring of ICH treatment (Figure 5E). Engineered cell technology is a powerful means in the treatment of ICH.

**FIGURE 6:**
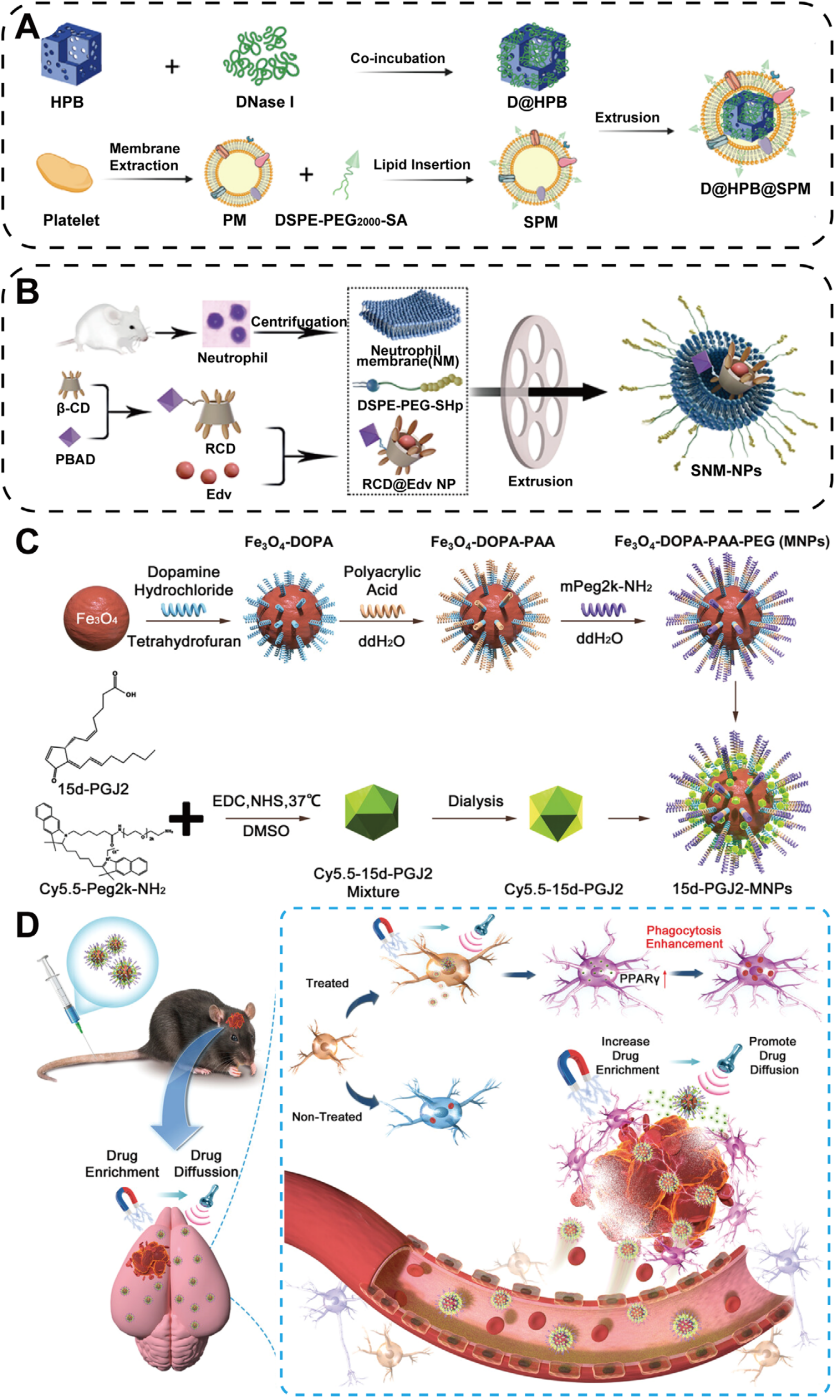
Novel nanotechnology for targeted treatment of nerve damage. (A) Sialic acid (SA)‐modified platelet membrane shell and hollow PBNPs core carrying DNase I constitute D@HPB@SPM [182]. Copyright 2024, Wiley‐VCH GmbH. (B) Schematic representation of SNM‐NPs, the system has multiple step‐by‐step targeting capabilities [183]. Copyright 2023, Wiley‐VCH GmbH. (C) Schematic representation of magnetic nanoparticle synthesis. (D) 15d‐PGJ2‐MNPs respond to magnets and focused ultrasound targeted hematoma for ICH [185]. Copyright 2023, Wiley‐VCH GmbH.

Functional nanoparticles have various modification methods. They can be attached to the cell membrane or encapsulated within the cell to construct a multiresponsive system, thereby achieving stepwise release. By applying the concept of biomimicry, nanotechnology can further enhance the therapeutic effect through responsiveness and specificity [181]. The emergence of nanozyme concepts has also broadened the therapeutic approach. Song et al. [182] designed a biomimetic nanozyme coated on the neutrophil membrane (NM) to reduce the damage caused by postthrombolysis brain hemorrhage (Figure 6A). Prussian blue nanoparticles (PBNPs) have a similar role to endogenous antioxidant enzymes and are considered effective ROS scavengers. Incorporating deoxyribonuclease I (DNase I) within PBNPs particles can exert antioxidative effects and prevent reperfusion brain hemorrhage. Importantly, the DSPE‐PEG2000‐SA‐modified NM (SPM) could more effectively deliver the encapsulated the D@HPB to the injured brain area and alleviate the oxidative stress injury to a greater extent.

In order to fully utilize the multilevel stepwise targeting capabilities, Dong et al. further modified the membrane of NMs with stroke homing peptide (SHp), enabling a faster targeting to damaged neurons (Figure 6B) [183]. This biomimetic nanodelivery system (SMN‐NPs) can penetrate the BBB and exert a targeted effect. Neutrophils target the inflammatory areas, and SHp targets the damaged neurons. Encapsulated within are indomethacin (Edv) and β‐cyclodextrin (RCD), which respond to ROS, and further regulate Edv release for synergistic ROS elimination, inflammation inhibition, and damaged neuron microtubule repair. The designed and synthesized biomimetic nanocarriers targeting hematoma improve the therapeutic effect of ICH and help restore neurological function.

Although the biomimetic cell membrane strategy relies on the natural targeting ability of the cell membrane to the inflammatory site, other approaches attempt to further improve the targeting accuracy through exogenous physical means. A novel nanotechnology combining magnetic‐targeted nanocarriers with focused ultrasound for treating ICH has emerged [184]. Whether the combination of the two can achieve better neuro regenerative effects after ICH was explored in a 2023 study [185]. The surface modification of iron oxide nanoparticles (MNPs) and PPARγ agonist (15d‐PGJ2) was designed and synthesized to have good magnetic and biocompatible nanoparticles (15d‐PGJ2‐MNPs) (Figure 6C). In vitro experiments confirmed the targeted brain delivery capability of the nanoparticles in response to magnets, and the use of focused ultrasound further increased the permeability of BBB. Experimental results showed that the combined use of two techniques enhanced clearance of hematomas by macrophages, alleviated neural inflammation, and the behavioral tests of the mice after treatment revealed significant improvement in neurological function deficits (Figure 6D). The emergence of this method highlights the clinical potential of combining focused ultrasound with nanotechnology in targeted hematoma treatment. This biological hybrid approach offers a promising solution for precise treatment of ICH.

Biomimetic targeted nanotechnology is emerging as an innovative drug delivery platform for stroke treatment with its broad applicability [186]; it can effectively cross the BBB and deliver drugs to the site of brain injury through thoughtful design and modification. Moreover, the application of biomimetic principles enhances the nanotechnology's targeting capability while minimizing adverse reactions. Importantly, this approach facilitates the integration of multiple treatment strategies into a single system, thereby exerting a multitargeted therapeutic effect.

#### 4.3.2 Neural Tissue Engineering

In addition to hematoma‐responsive drug delivery, advanced biomaterials can also provide neuroregenerative structures and spaces after ICH. These engineered structures can facilitate the migration and differentiation of endogenous neuronal cells into the damaged brain area, provide functional integration, and also serve as excellent carriers of therapeutic drugs for multiple therapeutic effects [187].

**FIGURE 7:**
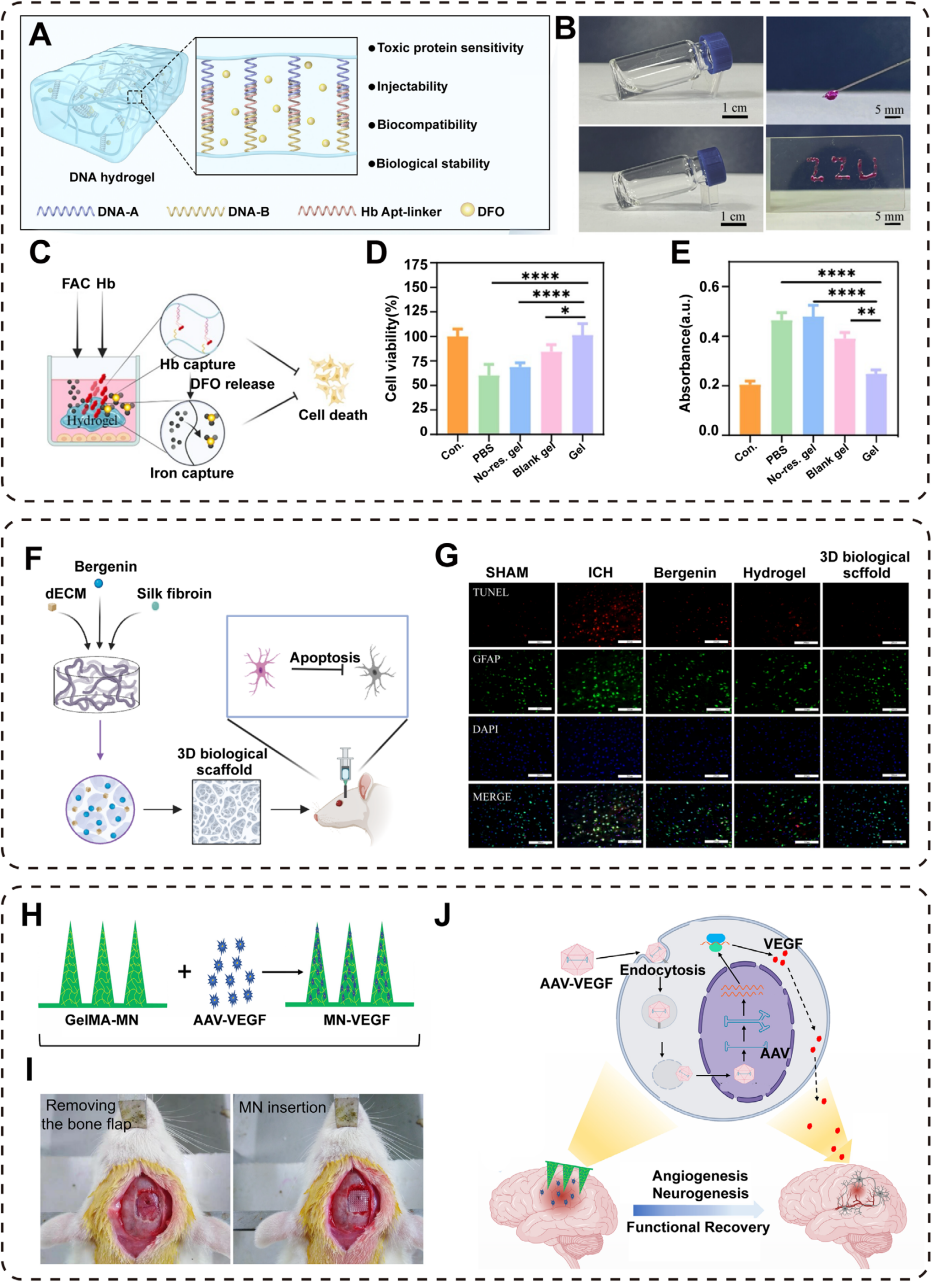
Neural tissue engineering materials promote nerve repair at hematoma site. (A) Schematic representation of injecting biomaterial into ICH lesion [190]. (B) Image of coagulation process and injectivity of hydrogel. (C) Schematic diagram of protective effects of hydrogels against ferroptosis and hemoglobin toxicity. (D) Cell viability was measured by CCK‐8 after treatment with ferric ammonium citrate (FAC, 150 µM) and hemoglobin (100 µM). (E) LDH activity detection of HT‐22 cells via LDH assay. Copyright 2024, American Association for the Advancement of Science. (F) Bergenin's 3D biological scaffold synthesis and mechanism in ICH therapy. (G) Immunostaining images of TUNEL staining results of different groups of rat brain sections [199]. Copyright 2024, Springer Nature. (H) VEGF‐overexpressing AAV was loaded into gelma‐based MN patches (GelMA MNs). (I) MNs were applied to the rat brain. (J) The process of MNs delivery into the ischemic brain and the sustained release of AAV vectors overexpressing VEGF [206]. Copyright 2021, Elsevier B.V.

Injectable hydrogels have unique advantages in the treatment of ICH, as it can inhibit ferroptosis of cells [188, 189]. In this background, Zhang et al. [190] utilized an hemoglobin aptamer as a DNA cross‐linking agent to connect acrylamide, forming a DNA hydrogel that encapsulates DFO (Figure 7A). The hydrogel has a better performance of the original flavor injection. In the presence of hemoglobin, the aptamer will bind to the target, triggering hydrogel degradation and controlled DFO release, which can realize the self‐treatment by inhibiting ferroptosis in neuronal cells (Figure 7B). To verify the neuroprotective effects of this hydrogel, in vitro experiments were conducted (Figure 7C). After treatment with ferric ammonium citrate (FAC) and hemoglobin, cell viability was determined using CCK‐8 and lactate dehydrogenase (LDH) (Figure 7D,E). The results confirmed that the hydrogel could effectively inhibit ferroptosis and exhibited strong a strong neuroprotective effect. Similar to this study, Zhu et al. [191] developed an innovative thermosensitive gelatin hydrogel specifically for repairing neural damage caused by iron injury after ICH. By adding DFO to the hydrogel, it can promote faster adsorption of iron in the hematoma area, reduce iron overload, and thereby protecting neurons [192].

In addition to serving as drug delivery carrier, advanced hydrogels exhibit characteristics similar to the natural extracellular matrix (ECM), providing the necessary structural and biochemical environment for the neural repair process [118]. The three‐dimensional (3D) microstructure of hydrogel provides a suitable structure for cell growth, axonal extension, and vascular reconstruction, which are key processes for functional neural tissue regeneration [192, 193]. Therefore, Bolan's team further explored the feasibility and safety of injecting hydrogels during the chronic phase of ICH [194]. They used a novel self‐assembling peptide hydrogel (SAPHs) to study its impact on neural recovery after ICH. Transcriptional analysis showed that SAPHs implantation did not trigger adverse inflammatory response or neurotoxicity, and it promoted cell proliferation at the implant site. Hydrogels may have the potential to serve as a delivery platform for other regenerative therapies [195]. Some studies have mixed injectable hydrogels with growth factors, which can promote the proliferation and migration of endogenous nerve cells [196]. Thus, it can enhance the recovery of neural function. In the recovery stage after ICH, the use of hydrogels can fill the cavities, induce the proliferation of endogenous NSCs, promote the recovery of neural function, and create favorable conditions for cell proliferation and migration.

Hydrogels play a role as a crucial “bridge” in the process of nerve repair. The concept of biological scaffolds has been developed based on hydrogel, leading to scaffolds becoming both therapeutic and delivery systems for nerve cell regeneration in clinical studies of ICH [197]. Additionally, it is interesting to note that the design concepts of hydrogels and scaffolds are becoming more and more similar, reflecting the common goals of biomimetic, bioactivity, and integration with host tissues [198]. The following sections discuss the recent progress of scaffold‐based ICH repair strategies, emphasizing their structural and functional contributions in neural tissue engineering.

In order to improve biocompatibility and degradation of traditional hydrogels, 3D‐printed bioscaffolds also have strong potential for the treatment of ICH [199], and the research on its scaffolds is also considered to be a further development in the field of hydrogels. The biological scaffold has excellent drug‐loading capacity and spatial structure, which is more conducive to nerve repair. The use of decellularized ECM can simulate natural tissues, enhance cell adhesion, and the filipin proteins stabilize scaffold structures. Additionally, the encapsulation of natural compounds such as bergenin has anti‐inflammatory and antioxidant properties (Figure 7F) [199]. And the results of TUNEL staining proved that the use of this biological scaffold in the acute stage could significantly reduce cell death and alleviate brain injury in ICH rats (Figure 7G). Considering the therapeutic window for treating ICH, scaffolds are often used during the recovery period to promote endogenous neural repair [200]. Neurofunctional disorders after ICH often result from inadequate promotion of neural cell proliferation and differentiation after secondary brain injury. Thus, scaffold design is believed to enhance the recovery of neurological after hematoma removal. Zhang's team developed a 3D, biodegradable, water‐based polyurethane scaffold for brain tissue regeneration after ICH [201]. This 3D porous polyurethane scaffold has pores of appropriately sized, which is conducive to the migration of NSCs.

Although microneedles (MNs) technology has not yet been directly applied to the treatment of ICH, its effectiveness in delivering drugs to the central nervous system and exerting therapeutic effects indicates its significant translational potential [202]. MNs provide a minimally invasive, controllable, and localized drug delivery method, and this technology can penetrate the BBB, which is the main challenge in treating brain injuries [203]. Recent studies have shown that the MNs technology has successfully been used to treat glioblastoma and ischemic stroke by directly delivering chemotherapy drugs, viral vectors, and growth factors into brain tissue [204, 205].

For example, Liu et al. designed a GelMA MNs loaded with an adeno‐associated virus (AAV) expressing human VEGF (AAV‐VEGF) (Figure 7H) [206], which can be used to continuously release AAV‐VEGF, promoting angiogenesis and functional recovery after ischemic stroke (Figure 7I,J). Similarly, silk fibroin MNs loaded with multiple drugs have shown promise in treating brain tumors through a delivery mechanism of precise targeting and responsive release. Given the common pathological features of these neurological diseases, such as inflammation, oxidative stress, and neuronal apoptosis, the MN technology could be rationally designed for the treatment of ICH. It can deliver neuroprotectants, anti‐inflammatory compounds, or stem cell‐derived exosomes directly to the area around the hematoma, thereby improving neurological recovery.

Moreover, the personalized design of the materials and shapes of MNs can fully exert the loaded drugs according to different implantation depths and release dynamics to adapt to the spatiotemporal characteristics of ICH injuries. With the continuous deepening of research, MNs have important prospects as next generation of drug delivery tools for neurological diseases and deserve further exploration in preclinical ICH models.

### 4.4 Neuroelectronic Interfaces

Research on treatment strategies for ICH focuses on targeting the pathogenesis, aiming to prevent the expansion of damage at its root, further save neural cells, and ensuring subsequent neurofunctional recovery [207]. However, due to the current limitations in treatment, the disability rate resulting from stroke remains relatively high, imposing a heavy burden on both families and society. The emergence of BCI technology provides a new approach to minimizing damage to the motor system [208]. By directly converting the patient's neural signals related to movement into external motor feedback, this technology has garnered significant clinical attention and is considered one of the top ten areas of focus in the field of biomedicine for the next decade. More importantly, the introduction of this technology fills the gap in critical early treatment options after stroke.

#### 4.4.1 Closed‐Loop BCI Systems

BCI technology has shown significant potential and promise in rehabilitating various neurological diseases including ICH [209]. As previously discussed, research in this field aims to rescue neural cells, as their proliferation and differentiation are closely associated with the degree of neurological recovery. Currently, clinical treatment methods lead to around 77% of stroke patients experiencing some level of motor or language disorders [210]. Due to the distinct functional regions of the brain, stroke frequently causes a substantial loss of motor control, with severe instances leading to hemiplegia and bed confinement. The recovery of neurological function poststroke is crucial, with active engagement in rehabilitation exercises and prolonged use of affected limbs proven to enhance flexibility. Nevertheless, current gold standard treatment plan is only suitable for ICH patients who still have some degree of motor ability [211]. Effective treatment methods are still absent for individuals with severe secondary damage and an inability to generate motor abilities. Thus, the emergence of BCI technology provides an advanced treatment method, by identifying brain nerve signals to connect the human brain with external devices for controlling actions, thereby accelerating neurological recovery [212].

Patients with severe stroke usually lack the minimum motor ability required to start routine rehabilitation training immediately after clinical treatment, which hinders their immediate participation in neurological function recovery training. BCI technology has become a promising tool for promptly conducting such rehabilitation exercises [213]. Motor dysfunction caused by stroke is a primary target for postoperative neurological recovery. BCI systems are frequently combined with three output feedback units: functional electrical stimulation (FES), robotic systems, and virtual reality (VR). Currently, BCI–FES system technology is more mature and widely used in neurorehabilitation training after stroke, with also ongoing clinical applications [214]. Biasiucci's team evaluated that BCI–FES therapy could generate stronger functional recovery effects for chronic stroke patients with motor impairments [209]. The findings revealed that FES intervention could reactivate specific muscle groups and markedly enhance muscle strength following neural stimulation. Similarly, the results indicated a relationship between functional plasticity and cortical reorganization in the brain. Current research suggests that BCI may be used to train and stimulate brain activity, thereby helping stroke patients regain their motor functions.

**FIGURE 8:**
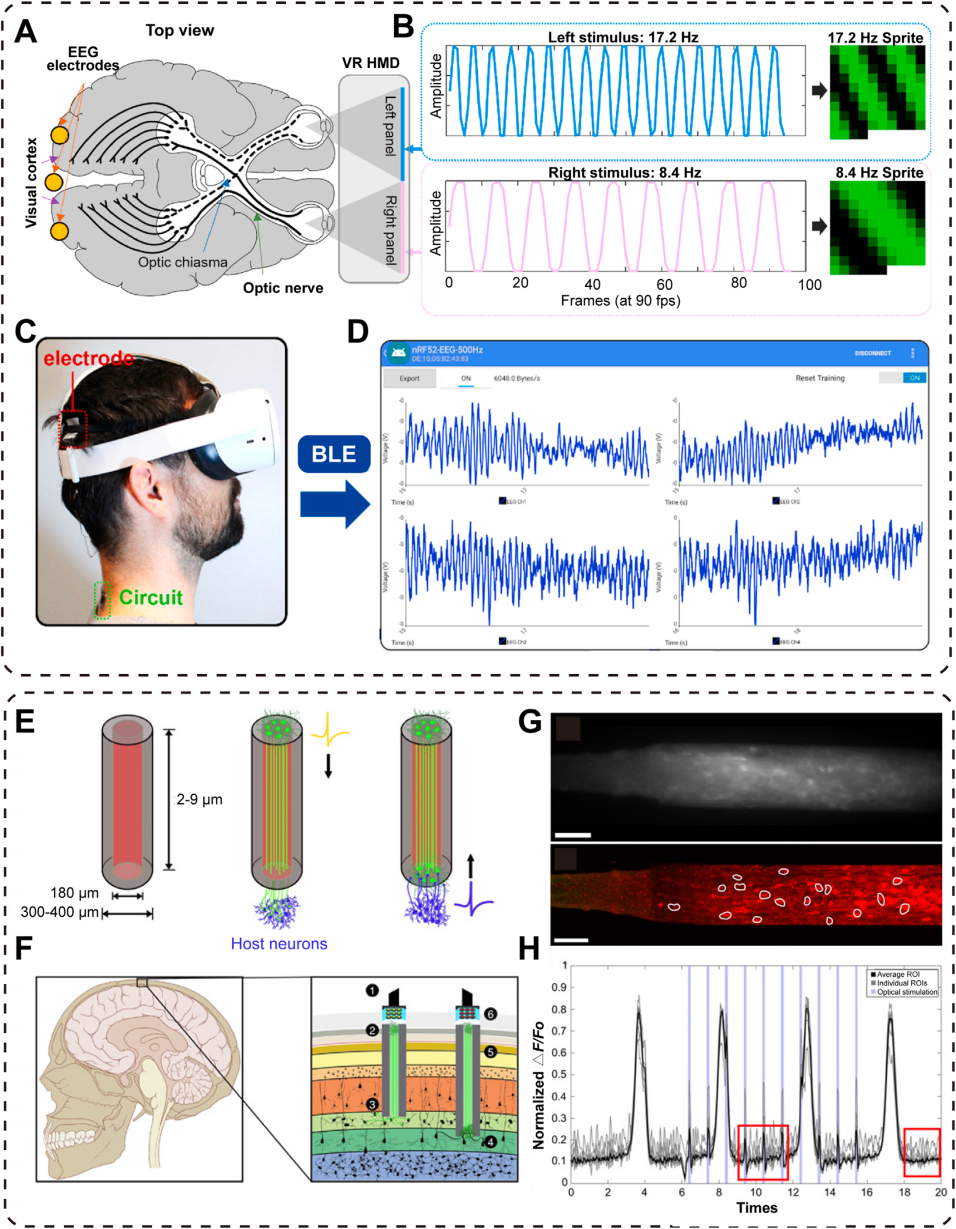
Emerging BCI technology improves dysfunction after nerve injury. (A) A cross‐sectional top view of the relationship between the optic nerve and the cerebral cortex and the VR head‐mounted display [215]. Copyright 2022, Elsevier. (B) Asymmetric stimulation can increase the information data processing capacity of portable BCI. (C) Photo of the subject wearing a VR device supported by a wireless soft bioelectronic system. (D) The data captured by the four EEG channels are transmitted to the central processing unit for processing via BLE. (E) The size of the implantable neuroactive electrode [234]. Copyright 2021, American Association for the Advancement of Science. (F) The photogenetic µTENNs active electrode can activate the host neuron through the light stimulation input signal of the light‐emitting diodes array, and the transmitted neuron neurons will be recorded by the photodiode array on the brain surface. (H) Normalized pixel intensity of ROIs in the neuron aggregates during the stimulation period. The black solid line represents the overall average ROI, and the µTENNs activities during and after the stimulation are circled in red.

The advancement of robotic systems and VR technology has significantly broadened the applications and enhanced the therapeutic efficacy of BCI systems [215]. By designing unique stimuli of different frequencies and lightweight soft contact electrodes, a new type of portable BCI device supporting VR has been developed (Figure 8A) [215]. Based on the asymmetry of the connection between the sensory receptors and the visual cortex of each eye, the development of asynchronous stimulation modes can improve the ability of the BCI device to analyze information (Figure 8B). And the signals of the four data recording channels are transmitted to the central processing unit for data analysis through Bluetooth Low Energy (Figure 8C,D). Compared with the traditional BCI technology, it has unique advantages in detecting brain signals. This portable BCI equipment is more conducive to daily neurological function rehabilitation.

In addition, BCI technology has also been developed for language rehabilitation after stroke, providing patients with a cost‐effective solution [216]. BCI technology can directly output signals from the cerebral cortex as text on a display screen, providing another communication tool. Moreover, this technology can also enhance the remodeling of neuronal functions by stimulating specific language circuits in the brain. Tang et al. [217] has designed a noninvasive language decoder based on BCI technology, which records the meaning in the cerebral cortex using functional magnetic resonance imaging. The language decoder can regenerate easily understandable language, providing a new approach to the recovery of the language system after stroke. Proix et al. [218] has demonstrated that high‐frequency activities in the brain are beneficial for language encoding by sampling and analyzing patients’ cortical electroencephalogram (EEG) signal data for language encoding. The application of BCI technology for vagus nerve stimulation has also been studied and shown to be a potentially effective therapeutic strategy for regulating language therapy systems [219].

The research overall indicates that BCI has the potential to facilitate stroke recovery by targeting sensory abilities, motor imagery, language deficits, and the integrity of the BBB. Subsequent studies might concentrate on enhancing BCI systems for personalized and efficacious neurorehabilitation after stroke.

#### 4.4.2 Biohybrid Interfaces

Brain hemorrhage leads to the death of nerve cells and the formation of glial scars, which hinder the recovery of neurological function. The biohybrid method can take advantage of the strengths of biological and synthetic systems to achieve more complex neurological function recovery effects [220, 221]. The integration of bioactive materials and electronic components produces a new type of biohybrid neural functional interface. Compared with traditional invasive electrodes, the biohybrid functional interface can enhance biocompatibility and specificity [222]. Conductive hydrogels are considered a promising BCI material [223, 224]. Hydrogels can be modified to change their mechanical properties to better adapt to human tissues, and adding multifunctional components for drug delivery, cell culture [225], and other applications [226], thereby achieving greater flexibility and therapeutic effects than single electrodes.

As previous studies have shown that implanting a suitable hydrogel carrier can enhance the neurological recovery of ICH. Inspired by biomimetic hydrogels, a bioadhesive brain–machine interface device has been developed by combining hydrogels with flexible microcircuits [227]. The functionally integrated bionic hydrogel flexible electrode can be implanted on the surface of the cerebral cortex to monitor the intracranial electrocortical map (ECoG) [228]. Wang et al. [229] inserted dopamine methacrylate‐hybridized poly (3,4‐ethylenedioxythiophene) nanoparticle (dPEDOT NP) into a network of carrageenan and PDA–polyacrylamide to create a conductive biomimetic hydrogel. The hydrogel has low modulus and high adhesion and can achieve a good fit with brain tissue. The performance was tested by attaching the bioadhesive ultrasoft BMI to the dura mater on the primary visual cortex in the left hemispheres of a rat brain. The results showed that the SNR value (10.19 dB) of the bioadhesive and conductive hydrogel integrated BMI was significantly better than that of the metal electrode. This method significantly improved the signal quality and stimulation effect.

Although hydrogel‐based bioelectronic devices hold great promise, their function can be compromised after implantation due to degradation caused by the abundant cerebrospinal fluid [230]. Current research has proposed biomimetic neuromorphic devices, which can function normally in high‐humidity environments and can be better implanted in the brain to create neuroprosthetic [231]. And some protein‐based biomaterials have been used to fabricate biocompatible artificial synaptic devices. Biomimetic neural synaptic devices constructed from bioactive materials exhibit greater stability than those based on traditional organic materials [232]. Gelatin–PEDOT:PSS composite materials have high electrical conductivity and flexibility [233]. Using them as active layers can produce synaptic behavior under high humidity conditions. The advancement of this biomimetic synaptic devices offers a promising therapeutic strategy for promoting neurological recovery after stroke.

The combined optical stimulation and gene‐modified optogenetic interfaces provides unprecedented cellular specificity for neural regulation after ICH. By constructing network‐level architectures that mimic the brain, this method facilitates the reconstruction of functional neural circuits. Cullen et al. [234] connects discrete neuronal populations via long axonal tracts to form microtissue‐engineered neural networks (µTENNs). The µTENNs were encapsulated in hydrogel cylinders and further developed into implantable axon‐based conduits (Figure 8E). The µTENNs are implanted at a predetermined depth to form synapses with local neural circuits and to propagate information along its axons to and from an external apparatus at the brain surface (Figure 8F). Moreover, genetic modification of neurons prior to implantation enables the expression of optogenetic proteins, thereby allowing optically driven neural regulation. Functional connectivity was confirmed through optical imaging of GCaMP signals after implantation (Figure 8G,H). The “living electrodes” proposed in this study may provide a new treatment strategy to replace traditional microelectrodes and lay the foundation for the development of new neural interface devices based on µTENNs.

Furthermore, the fate of stem cells during neural development can be guided by a combination of chemical [235], mechanical [236], and electrical stimulation [237]. Several reports confirmed that the application of specific growth factors or modifications to external physical conditions can promote the differentiation of stem cells into different type of neural cells with distinct functions. Chung et al. [238] designed a microfluidic chip with neatly arranged microelectrodes on its surface. By culturing iPSCs in a microfluidic chip, applying electrical stimulation and neurotrophic factors can significantly improve the efficiency of human iPSCs in promoting the differentiation of functional neurons and enhance synaptic interactions [239]. The synergistic effect of electrical and biochemical stimulation is a promising strategy for treating neurological dysfunction after ICH.

However, these methods face some challenges, including the need to increase the production of mature and functionally differentiated neurons and to overcome immune responses triggered by implantation [240]. Appropriate clinical studies are needed to prove the role of neuroelectronic interfaces in neurological rehabilitation.

## 5 Clinical Translational Value

Convincing evidence from preclinical studies indicates that enhanced neurogenesis and improved functional outcomes after ICH have generated considerable impetus for clinical translation. Table 2 provides an overview of these emerging approaches, outlining their characteristics, mechanisms of action and therapeutic effects. But implementing innovative therapeutic options in ICH research into clinical trials to enhance patients’ neurological function remains a significant problem. Even though the research has produced many encouraging scientific findings, the process of clinical transformation is still long and complex, and many obstacles must be addressed. Utilizing advanced biomarkers to assess patient classification, improve multitarget combination therapy strategies, and assess the safety of intervention measures in the biomedical area is essential to accelerating the clinical transformation of ICH treatment strategies.

**TABLE 2:** Novel therapeutic strategies for nerve regeneration.

Treatment strategy	Effective components	Feature	Therapeutic effects	Year	References
Cell transplantation	MSCs overexpressing GDNF	Reducing the bleeding volume	Promoting angiogenesis and the survival of neurons	2023	Jiang et al. [107]
	NSC overexpressing miRNA‐21	Targeting SOX2 and disrupting the SOX2/LIN28‐let‐7 axis	Enhancing nerve regeneration and functional recovery	2025	Dai et al. [241]
	Artificial cells loaded with mitochondria	Magnetic targeting and promoting microglial immune homeostasis	Reducing cerebral edema and promote better survival of nerve cells	2025	Zhou et al. [242]
	Adipose‐derived pericyte	Neuroinflammatory regulation and repair of BBB	Reducing neurological dysfunction	2023	Zhang et al. [243]
Cell engineering technology	Neutrophil membranes and edaravone	Targeting inflammation	Enhancing the phagocytosis of red blood cells and adjusting the inflammatory microenvironment	2024	Dong et al. [183]
	Platelet membrane and polydopamine	Targeting bleeding sites	Reducing oxidative stress and repairing damaged blood vessels	2023	Xu et al. [244]
	Neutrophil membranes and nanozymes	Targeting hemorrhagic sites and inhibiting ROS production	Improving the neuroinflammatory microenvironment and facilitating behavioral recovery	2025	Xu et al. [245]
	Peptide‐modified engineered nano erythrocytes	Targeting microglia	Regulating the phenotype of microglia and immune response	2022	Na et al. [246]
	Macrophage membrane and angelica polysaccharide	Targeting inflammation	Adjusting the inflammatory microenvironment	2022	Su et al. [247]
Nanotechnology	Polyphenol nanoparticles	Chelating iron and reducing oxidative stress	Increasing survival rate and improving neurological function	2025	Zeng et al. [248]
	Pterostilbene nanoparticles	Intranasal administration targets	Alleviating brain injury and neurological deficits	2025	Duan et al. [249]
	Platinum nanozyme	Inhibiting the NF‐κB pathway	Reducing inflammation and eliminate ROS	2025	Guo et al. [250]
	Prussian blue nanozyme	Targeting mitochondrial and ROS responses	Alleviating oxidative stress and reducing cell apoptosis	2023	Liu et al. [251]
	Bioinspired manganese‐organic framework	Dual anti‐ROS effect	Eliminating ROS and enhancing angiogenesis	2023	Jian Wang [252]
	Peptide‐templated manganese dioxide nanozyme	Targeting brain thrombi and extending half‐life	Inhibiting the secretion of proinflammatory factors	2023	Wang et al. [253]
	IL‐10 liposomes	Targeting hematomas	Accelerating hematoma absorption	2023	Han et al. [176]
	Diosmetin liposomes	Targeting hematoma and regulating microglial cell polarization	Anti‐inflammatory	2025	Gu et al. [254]
	Idebenone micelle	ROS response	Inhibiting neuronal ferroptosis and inflammation	2023	Li et al. [255]
	Resveratrol micelle	pH‐sensitive response and targeting Mitochondria	Alleviating ROS and regulating microglia phenotype	2023	Wang et al. [256]
Scaffold	Porous 3D polyurethane scaffolds	Regulating cell phenotype	Promoting endogenous brain regeneration	2023	Zhang et al. [201]
	MSCs scaffold	Improving cell survival	Promoting the recovery of neurological function	2022	Takamiya et al. [257]
	3D‐printed exosome scaffolds	Increasing adhesion of NSCs	Promoting nerve repair	2023	Liu et al. [258]
Hydrogel	Self‐assembling SAPH peptide hydrogel	Injectable and biomimetic	Promoting the recovery of neurological function	2023	Bolan et al. [194]
	3D printed exosome hydrogel	Regulate the activity of pathological MMPs and the expression of inflammatory cytokines	Promoting the regeneration of nerve tissue and reducing edema around hematoma	2025	Zhang et al. [259]
	H_2_S hydrogel	Sustained drug release in situ	Reducing brain edema	2022	Chen et al. [260]
	3D printed ECM hydrogel	Promoting cell migration through the Slit2–Robo1 pathway	Promoting the recovery of neurological function	2025	Xia et al. [261]
	NSCs and human umbilical vein endothelial cells	Promoting ECM reshaping	Promoting endogenous nerve cells	2025	Kim et al. [262]
	Curcumin and edaravone hydrogel	Asynchronous dual‐drug delivery	Promoting neurogenesis and angiogenesis	2023	Lin et al. [263]
Neuroelectronic interfaces	BCI–FES	Functional electrical stimulation	Improving the exercise outcomes of stroke patients	2024	Catherine et al. [264]
	BCI for motion imagery	Motor imagery training	Promoting the recovery of upper limb function after stroke	2024	Xu et al. [265]
	Biohybrid device	Cell and flexible electrode functions are integrated	Enhancing neural function repair	2023	Damiano et al. [266]
	Biohybrid neural interface	Synaptically stimulating deep brain targets	Promoting the growth of nerve axons	2025	Sifringer et al. [267]

### 5.1 Biomarker‐Driven Stratification

Different stages of ICH progression have different characteristics. Through the understanding of the pathophysiological cascade of ICH, clinical treatment strategies need to shift from simple modeling to patient personalization. Biomarker‐driven stratification of ICH severity in patients has important guiding significance for the subsequent adoption of corresponding treatment strategies and can maximize the treatment effect of different types of patients.

#### 5.1.1 Multiomics Signatures

Mining through data such as genomics, proteomics, and metabolomics can reveal the key biomarkers that reflect the complex biological characteristics of the pathophysiology of ICH. By identifying and verifying them, it is crucial for the early diagnosis, prognosis assessment, and treatment response of ICH. Proteomic analysis shows that specific brain‐derived proteins released into the blood or cerebrospinal fluid have important reference value for indicating the clinical outcome of cerebral hemorrhage. Studies have shown that glial fibrillary acidic protein (GFAP) is a marker of astrocyte activation or damage [268]. The detection of this indicator may reflect the hematoma volume and the severity of neurological damage, so it is of great significance for the early diagnosis of ICH. Ubiquitin C‐terminal hydrolase L1 (UCH‐L1) is a neuronal protein that is rapidly released after neuronal injury and is related to the disruption of the BBB. Therefore, it can be used to distinguish ICH from ischemic stroke and assess the severity [269, 270]. The application of UCH‐L1 in clinical practice is also increasing. Recent studies have shown that simultaneously detecting the expression levels of UCH‐L1 and GFAP in the blood can improve the accuracy of diagnosis. In addition, neurofilament light chain, which is also considered a sensitive biomarker for various neurological diseases and the severity of neuronal injury, and its level in blood is related to the long‐term prognosis of stroke [271]. By utilizing the level of these biomarkers, doctors can further divide patients groups into different types of brain injury and make specific prognoses and implement targeted treatment accordingly.

#### 5.1.2 Wearable ICP Monitoring Devices

Standard ICP monitoring methods rely on invasive techniques, including the use of intraventricular catheters or brain parenchymal probes. Although these methods provide accurate information for timely treatment of ICH, they involve risks of bleeding and infection [272] and are not suitable for all patients. In recent years, with the emergence of noninvasive wearable ICP monitors, safer and more convenient monitoring has become possible [273].

The continuous development of transcranial Doppler ultrasound technology can continuously monitor ICP by measuring pulse index and evaluating optic nerve sheath diameter [274], which provides conditions for the development of noninvasive and portable ICP monitoring devices. These devices utilize machine learning algorithms trained on invasive ICP measurements to provide accurate noninvasive predictions. In addition, pupillometry or optical methods based on near‐infrared spectroscopy have also been used to estimate ICP changes [275, 276]. Wearable devices are combined with cloud‐based analytics, they can enable remote patient monitoring, early deterioration alerts, and personalized treatment adjustments. The portability of the device can also be extended to daily monitoring use, thereby improving patient treatment compliance. Clinical implementation of wearable ICP monitoring devices faces several challenges, including accuracy compared with invasive measurements, the standardization of alarm thresholds, and integration with hospital information systems. In addition, more randomized controlled trials are needed to determine the reliability of continuous ICP monitoring.

### 5.2 Combination Therapy Optimization

The complex pathophysiological mechanism of ICH determines that its treatment strategy needs to be multifaceted, and multiple therapeutic effects should be exerted on different pathways involving inflammation, oxidative stress and excitotoxicity [277]. Current treatment methods have shown that the therapeutic effect of a single pathway is limited. Combination therapy or multilevel targeted therapy targeting multiple harmful mechanisms is expected to have greater hope in synergistic neuroprotection and improving patient prognosis, but these strategies need to be examined from multiple aspects.

#### 5.2.1 Sequential vs. Concurrent Strategy Design

The intricate pathophysiological causes of ICH require multimodal treatment strategies. Improving patient outcomes still depends on figuring out when interventions should be implemented and creating efficient combinations of multitarget therapy techniques.

It is crucial to conduct a methodical assessment of both concurrent and sequential therapy approaches. In the acute phase of ICH, priority is given to controlling blood pressure to stop bleeding and preventing the expansion of hematoma [278]. The drugs currently studied, such as recombinant factor VII or other hemostatic biomaterials, mainly reduce acute inflammatory responses in the subacute phase, and finally promote neuroprotection in the chronic phase, emphasizing neurorepair and regeneration [279]. In order to improve prognosis of ICH patients, multitarget treatment strategies have been developed. Concurrent strategies usually involve the simultaneous use of multiple therapies, aiming to achieve potential synergistic effects by targeting multiple pathological processes from an early stage [280]. The new biomedical strategies currently under study focus on multiple therapeutic purposes that can play an anti‐inflammatory and enhanced neurogenesis. Sequential or concurrent treatment strategies should also consider the kinetic characteristics and interactions of between drugs in experiments and achieve multiple therapeutic effects by determining the targeting of different pathological mechanisms and optimal treatment window. Additionally, the selection of combined treatment plans can be further optimized by monitoring ICP and neuronal injury markers, making the treatment strategy for ICH more accurately applicable to the patients.

#### 5.2.2 In Silico Trial Platforms

The complexity and high cost of clinical trials for ICH have hindered its drug development. The continuous advancement of computer‐aided drug design technology provides a feasible alternative for providing simulated pathological mechanisms and drug responses [281]. These platforms can integrate existing biological data, the kinetic or pharmacodynamic characteristics of drugs, and the digital model of ICH pathology, simulate and predict multiple treatment options in virtual patients on the computer to determine the most promising candidate treatment strategies for preclinical trials [282]. Moreover, this technology has made satisfactory progress in the field of cerebral ischemia, but it is still in the early stages of application in ICH [283]. Through computer, virtual patient simulation of clinical trials can help identify the most effective combination therapy and provide theoretical support for the design of future clinical trials. Although this technology is not yet mature at present, these platforms have the potential to significantly reduce the risks of ICH combination therapies and simplify their development pathways, making them more cost effective and efficient. In the future, with the improvement of computing power and the refinement of models, computer simulation clinical trials are expected to become an important tool for optimizing the treatment of ICH.

### 5.3 Regulatory Challenges

In response to the complex pathophysiological mechanisms underlying ICH, various of emerging multitarget treatment strategies have been designed and developed to achieve better neural recovery outcomes. Particularly, the combination of cell technology and novel biomedical platforms has brought about more complex and unique regulatory challenges. These challenges mainly arise from the advanced and complex nature of these treatment strategies and require further exploration of their potential risks and the improvement of current assessment standards.

#### 5.3.1 Cell Therapy Safety Concerns

In recent years, stem cell therapy has received great attention in the treatment of cerebral hemorrhage due to its potential to differentiate into neurons, replace damaged nerve cells, secrete neurotrophic factors, and promote axonal repair [128]. Many kinds of cells have shown significant improvement in neurological function in preclinical studies, but there are still major safety and regulatory barriers to overcome before clinical applications [284].

iPSCs and embryonic stem cells have attracted research attention, but they have the risk of uncontrolled differentiation, raising concerns about teratoma formation. To mitigate this risk, it is necessary to ensure complete differentiation into neural cells before transplantation, or to eliminate residual undifferentiated cells by using advanced purification techniques. In contrast, MSCs or fully differentiated autologous cells are generally the most studied types due to their low immunogenicity and minimal tumorigenic potential [285]. But there are still issues with stem cell survival, heterogeneity, and purity, particularly in the inflammatory and hypoxic microenvironment characteristic of acute ICH. In addition, long‐term monitoring data in patients are still limited, and the carcinogenic risk profile of these therapies is still uncertain.

Generally speaking, regulatory organizations for advanced therapies demand that the treatment be both safe and effective, that the source and quality of the cells be thoroughly assessed, and acknowledgment of the potential tumorigenic risks associated with cell therapy.

#### 5.3.2 Biomaterial Degradation Kinetics Matching

An increasing number of novel biomaterials can be used as carriers of drugs, stem cells, or bioactive molecules to develop new therapeutic strategies for ICHs. These strategies can enhance targeted delivery and regulate the hematoma microenvironment to promote neuroregeneration. However, even in the face of encouraging preclinical results, concerns about their biocompatibility and degradation kinetics remain major obstacles to clinical translation.

The brain environment reacts differently to the various stages of ICH, thus the biomaterials’ degradation kinetics must be in line with the pathophysiological requirements and treatment goals of ICH. This also poses a challenge to the clinical management of smart bioplatforms. Early degradation may cause the drug to be released prematurely and therapeutic effects lower than expected, while a prolonged presence places higher demands on the safety of the biomaterials. Therefore, it is essential that these biomaterials are nontoxic and that the body can effectively eliminate them once they have served their purpose, and it will be necessary to create future smart biomedical platforms with programmable degradation kinetics that can be matched to the ICH treatment window. Additionally, regulatory authorities must pay greater attention to the safety of intelligent biomaterials. For large‐scale production and clinical applications, the consistency between different batches is most important and should be confirmed through in vivo and in vitro safety and efficacy tests. Finally, he long‐term behavior after implantation must be verified through continuous monitoring to ensure the safety of patients and therapeutic effect.

Although various biomedical engineering strategies show great promise for neural repair after stroke, they clinical translation still face significant challenges and limitations (Table 3). Active collaboration between researchers and clinicians can help to improve the safety of novel therapeutic strategies and accelerate them into clinical trials. It is crucial for the clinical transformation of regenerative medicine by conducting more clinical trials and formulating reasonable and standardized treatment strategies.

**TABLE 3:** Advantages and challenges of intelligent biomedical engineering in the treatment of ICH.

Treatment strategy	Advantages	Limitations
Stem cell transplantation [286, 287]	Directly replenish nerve cells Accelerate neural network reconstruction	Effect uncertainty High cost Technical limitation Immunoreaction
Cell engineering technology [288, 289]	Targeted hematoma Good biocompatibility Protective nerve cell Enhanced endogenous nerve regeneration	Cytotoxicity Off‐target effects of gene editing
Nanotechnology [290, 291]	Targeted hematoma Through the BBB Multitarget therapy	Stability Immunoreaction
Hydrogel and scaffold [292, 293]	Provided a place for nerve regeneration Enhanced endogenous nerve regeneration Fill damaged brain tissue	Degradability Stability Safety
Microneedle [205, 294]	Targeted hematoma Controlled drug release Convenient and quick use	Drug loading property Penetration safety
Neuroelectronic interfaces [208, 295]	Stimulation of neural network connections Promoted neurological rehabilitation Improved patient outcomes	High cost Instrument complexity Long service period

## 6 Conclusion and Perspectives

Neural recovery and regeneration are a key factor influencing the prognosis of stroke patients. With the deepening of research on the pathological mechanisms of ICH, the focus of research has shifted from merely reducing mortality to repairing nerve damage. Emerging treatments in regenerative medicine have the potential to provide more options for the treatment of ICH. However, despite these advancements, converting promising preclinical studies into effective clinical applications remains a challenge. These gaps highlight the continuous need for precise classification of ICH patients and the development of advanced combined treatment strategies. The future direction of ICH treatment should aim to provide more opportunities for promoting nerve repair and conducting personalized treatment by integrating cross‐disease insights and advanced biomedical technologies.

### 6.1 Cross‐Disease Insights

The activation of microglia and astrocytes in ICH leads to the release of proinflammatory cytokines and ROS, which is similar to the inflammatory responses observed in ischemic stroke and traumatic brain injury (TBI) [296]. Therefore, immunomodulatory therapies are considered to be also applicable for reducing secondary brain injury in ICH. Furthermore, the neuronal necrosis and apoptosis observed in ICH are mechanistically similar to those observed in ischemic and TBI, suggesting that interventions targeting these death pathways may have cross‐disease applicability. New strategies for neuroprotection in ischemic stroke provide valuable ideas for the design of multitarget treatment for ICH, and related studies have verified them [297]. In addition, some biomarkers for the diagnosis, prognosis, or treatment monitoring of ischemic stroke and TBI may also have certain reference value in the treatment of ICH [298]. And BCI technologies can play a therapeutic role in neurodegenerative diseases such as Parkinson's disease and Alzheimer's disease by restoring neural circuits. It can also be used in the treatment of ICH to reconstruct damaged neural pathways and accelerate recovery after stroke [299]. Overall, drawing on the experiences from studies on ischemic stroke, TBI, and other neurological diseases can accelerate the development of ICH treatments.

### 6.2 Next‐Generation Technologies

The integration of intelligent medicine, precision medicine, and biomedical engineering has profoundly influenced the treatment system of modern medicine and is expected to revolutionize the diagnosis, treatment, and rehabilitation of ICH. This comprehensive strategy uses a variety of advanced technologies to meet the challenges of the complexity of ICH treatment. Advanced in vivo imaging technology and machine learning algorithms can provide new spatiotemporal insights for the clinical treatment of ICH through image analysis and data prediction and further improve the accuracy of prediction results by expanding patient data results. When combined with wearable sensor data for physiological monitoring, it can further enhance ICH diagnosis, promote patient stratification in clinical research, and enable clinicians to have more personalized treatment plans. In addition, artificial intelligence is expected to optimize clinical trial design and simulate and analyze the effects of combined treatments of multiple strategies through cloud databases. Intelligent biomedical engineering strategies can actively respond to the pathophysiological characteristics of ICH and carry out targeted therapy. Based on the rapid development of the information digital age, these technologies can provide comprehensive guidance for personalized treatment of ICH patients from digital analysis of the progression and prognosis of ICH, design of combined treatment strategies, and combined with intelligent biomedical engineering strategies. In the future, comprehensive technologies will continuously promote the clinical treatment of ICH and ultimately provide patients with personalized intelligent treatment systems.

### 6.3 Ethical Considerations

Ethical considerations are important in the development and application of new treatments for ICH. For treatment strategies with emerging cell therapies or smart biomaterials, involving high technical complexity, it is necessary to fully explain to the patients the possible adverse reactions or uncertainties and consider how to obtain adequate informed consent in cases where patients with acute ICH may fall into coma and other clinical reactions. Many emerging therapies are still in the early stages, and their clinical efficacy and safety are still unclear. Especially considering the tumorigenicity and instability of stem cell injection, the source of its cells and gene editing technology are also questioned by social ethics [300], and the irreversibility of intelligent biomaterials after implantation makes it crucial to conduct a long‐term safety assessment of them [301]. Before conducting clinical treatment, doctors should carefully weigh the benefits and risks of treatment based on the patient's own basic condition, and conduct long‐term follow‐up after the treatment [302]. In addition, the development of digital medicine requires the collection of more patient information for analysis, and the long‐term data collection will also raise concerns about privacy and data management. Multitarget treatment approaches have brought significant regulatory challenges; and clinical trials must adhere to strict ethical standards and emphasize the credibility of the experimental results, so that patients can have confidence in adopting new strategies for treating ICH.

In conclusion, the treatment of ICH is developing toward greater precision and personalization. By continuously deepening the understanding of the pathophysiological mechanisms of ICH, adopting various emerging strategies to accelerate neural repair, and address the strict challenges of clinical transformation, significant progress has been made in the precise treatment of ICH. Insights across disease mechanisms have accelerated the development of treatment strategies. Future technologies can enhance the accuracy and effectiveness of ICH treatment and significantly promote neural repair, bringing great hope for fundamentally improving the prognosis of patients.

## Author Contributions

Haojie Zhang: writing—original draft, conceptualization, and investigation. Yinping Pan: writing—review and editing. Liang Jin and Bochu Wang: review and editing, supervision, and funding acquisition. All authors have read and approved the final manuscript.

## Ethics Statement

The authors have nothing to report.

## Conflicts of Interest

The authors declare no conflicts of interest.

## Data Availability

No data were used for the research described in the article.
